# Human Immune System Reconstitution in NOD/Shi-*Prkdc^scid^ Il2rg^em1^*/Cyagen Mice to Study HIV Infection: Challenges and Pitfalls

**DOI:** 10.3390/life15071129

**Published:** 2025-07-18

**Authors:** Aleksey M. Nagornykh, Marina A. Tyumentseva, Aleksandr I. Tyumentsev, Leonid A. Fedotov, Konstantin S. Karbyshev, Evgeniya A. Orlova, Liliia N. Volchkova, Lubov S. Danilova, Andrey S. Akinin, Vasiliy G. Akimkin

**Affiliations:** Central Research Institute for Epidemiology of the Federal Service for Supervision of Consumer Rights Protection and Human Welfare, Moscow 111123, Russia; tyumentseva@cmd.su (M.A.T.); fedotov@cmd.su (L.A.F.); karbyshev@cmd.su (K.S.K.); evgeniia.orlova.msu@gmail.com (E.A.O.); danilova.l@cmd.su (L.S.D.); akinin@cmd.su (A.S.A.);

**Keywords:** HIV, mouse model, graft-versus-host disease, peripheral blood mononuclear cells, T-helper cells

## Abstract

The main challenge after engraftment of human tissues to mice is the development of graft-versus-host disease. It often occurs in an acute form, which reduces the time frame for observations. This is especially important to take into account when planning long-term studies of chronic diseases such as HIV infection. In addition, in mice, even with a similar genotype but different origin, the interaction between the graft and the recipient’s organism can manifest itself differently. We engrafted human immune cells in three different concentrations into immunodeficient NOD/Shi-*Prkdc^scid^ Il2rg^em1^*/Cyagen mice. Then, the initial points of development of a severe graft-versus-host reaction and the maximum possible time window for humane observation were determined. The study included regular complete blood count and the monitoring of the dynamics of the concentration of human cells in the blood of mice. In addition, the effect of grafts on the activation of the recipient’s immune system was assessed. Finally, necropsy and histological and immunohistochemical examinations of the organs were performed to determine the localization of human cells. In this way, critical factors determining the success of human immune system reconstitution in mice were identified.

## 1. Introduction

The importance of using animal models for research in a wide range of disciplines will not disappear for many years to come. In addition, the limitations of many animals for simulating human diseases have always been the main obstacle in studying pathologies, as well as developing means and methods for their therapy. Due to their variability, availability, and relative affordability, mice are at the top of the demand pedestal in animal research. At the same time, the development and advancement of new technologies provide us with advanced tools that make it possible to create models for specific purposes and tasks.

It is known that partially immunodeficient Nude mice are not suitable for the reliable restoration of the human immune system. However, Wei and co-authors used the novel CRISPR/Cas9 genome editing system to perform *Foxn1* deletion, creating hairless mice with the NOD/SCID/*IL2rg*^−/−^ genotype [1]. The development of such mice opens new possibilities for more humane research procedures and manipulations.

In general, the discovery of a spontaneous mutation in the gene encoding the protein kinase, DNA-activated, catalytic subunit (*Prkdc*) in CB17 mice served as a powerful stimulus for work on human cell engraftment [2,3]. This mutation partially blocks the development of mature T- and B cells, as well as the formation of endogenous immunoglobulins, providing severe combined immunodeficiency (SCID). However, the formation of mature natural killer cells does not provide long-term persistence of human cells in such mice. Deletion of *IL2rg* solves this problem. Therefore, SCID mice with deletion of *IL2rg* are more preferable objects for engraftment of human cells and tissues.

However, the persistence of a few functional immune system cells even in such immunodeficient mice requires preliminary immunosuppression. This is usually achieved by myeloablation using physical (irradiation) or chemical methods, or a combination of both [4].

The strain, age, and sex of mice directly affect the myeloablation protocol. Thus, the sublethal dose of irradiation for 6-week-old NOD-Prkdcem^26Cd52^ *Il2rg*^em26Cd22^/Gpt (NCG) mice is 1 Gy [5]. At the same time, for NCG mice on the background of NOD/Nju, the maximum sublethal dose was determined to be 2 Gy [6]. Therefore, the myeloablation protocol is one of the most important values in the humanization of mice.

Most animal studies need to mimic the immune system’s response to pathology. Several models of the human immune system have been developed, but three main types of grafts can be distinguished: human fetal organ fragments (e.g., bone marrow–liver–thymus, BLT), human hematopoietic stem cells (hHSCs), and human peripheral blood mononuclear cells (hPBMCs). All of these models have their own advantages and disadvantages. The BLT mouse model is considered the “gold standard” for studying the human immunodeficiency virus (HIV) [7]. Although this type of graft allows for the reconstitution of human lymphoid cells in many recipient organs [8], this model is difficult to perform.

hHSCs’ xenotransplantation induces the production of all mature blood cells and the reconstitution of human lymphoid tissue in some organs [9]. However, this model may develop a lethal phenotype as early as 84–126 days post-cell injection (d.p.i.) due to the proliferation and hyperactivation of human myeloid cells [10,11]. The hPBMC engraftment model is simple to perform and allows for earlier reconstitution of human cells, but there is also a risk of early development of xenogeneic graft-versus-host disease (GVHD) [12].

Another important factor is the concentration of xenografts. Thus, the introduction of 1 × 10^5^ and 5 × 10^4^ hCD34^+^-cells into NOD-*Prkdc^scid^ IL2rgtm1Wjl* (NSG) mice resulted in the reconstitution of 90% and 93% of circulating hCD45^+^-cells, respectively [13]. Of course, it should be taken into account that the mice that received 5 × 10^4^ hCD34^+^-cells were neonates. However, an increase in the antigen load of 50% allowed an increase in graft engraftment of only 3%. In earlier studies, administration of 7 × 10^5^ hCD34^+^-cells into NOD-*scid IL2Rγ^null^* mice resulted in only 6.3 ± 2.4% hCD45^+^-cells circulating in the peripheral blood but was considered successful [14]. It was reported that whole-body irradiation with a dose of 3 Gy was not lethal for these mice. Therefore, a correctly selected ratio of the myeloablation level and the graft concentration is the key to the success of the model.

Despite the fact that the use of immune system xenograft models is very popular, the clinical and morphological features of humanized mice are often not given attention. At the same time, many studies of HIV infection are long-term [8]. During this time, animals may develop pathologies that are not related to the study.

Therefore, the aim of this study was to compare the effects of engraftment of hPBMCs and hT cells mixed with autologous hPBMCs in NOD/Shi-*Prkdc^scid^ Il2rg^em1^*/Cyagen (C-NKG) mice over a long time. To this end, we determined the degree of human cells (HCs) engraftment (chimerism) and its effect on hematological parameters of recipients. In addition, we assessed the distribution of HCs in the mice. Finally, we determined the minimum duration of the study before the onset of severe GVHD symptoms. The data obtained here may help to predict the effectiveness of human immune reconstitution protocols at an early stage, which is especially important in HIV infection models. In the future, we plan to use the protocol we developed to humanize C-NKG mice for subsequent infection with HIV.

## 2. Materials and Methods

### 2.1. Animals

All manipulations were carried out under sterile conditions. Specific pathogen-free female 5- to 6-week-old C-NKG mice (Cyagen Biosciences Inc., Suzhou, China) were housed in the ISOcage P bioexclusion system (Techniplast, Buguggiate, Italy) at a temperature of + 21 to +23 °C and an air humidity of 45–65%. Each cage was equipped with an igloo-type shelter. Animals were maintained under a 12 h day/night cycle. Feeding was carried out with a standard maintaining diet for rodents (4RF21, Mucedola S.R.L., Settimo Milanese, Italy). The drinking was carried out with filtered tap water. Mice had unlimited access to water and feed. Fine-grained (3 mm fraction) birch chips (Russia) were used as bedding. Feed, drinking water, and bedding were sterilized in an autoclave before being given to the animals.

Animals were randomized into 7 groups of 6 mice per group using RandoMice v1.1.7 software based on body weight. RandoMice software was written in C# using. NET Framework 4.7.2 and Microsoft Visual Studio version 16.4 [15].

### 2.2. Cell Cultures

Two types of grafts were prepared for the study:

- 24 h hPBMCs after thawing and incubation in culture medium;
- Activated 10-day-old hT-cells mixed with autologous hPBMCs.

All grafts were isolated from the leukocyte concentrate obtained from a single healthy donor. The leukocyte concentrate was purchased from the Moscow Regional Blood Transfusion Station. Each type of graft was used in three different concentrations (Table 1). Control mice received a similar volume of 1× Dulbecco’s Phosphate-Buffered Saline (DPBS).

To isolate hPBMCs, the donor leukocyte concentrate was incubated with erythrocyte lysis buffer (155 mM NH_4_Cl, 12 mM NaHCO_3_, 0.1 mM EDTA, pH 7.4, 0.22 μm filtered). Unlyzed cells were pelleted by centrifugation and washed sequentially in 1× DPBS (21300-017; Gibco, Carlsbad, CA, USA) three times. hPBMCs were resuspended in fetal bovine serum (FBS, 2095ML100; NeoFroxx GmbH, Einhausen, Germany) supplemented with 10% dimethyl sulfoxide (786-1388; G-Biosciences, St. Louis, MO, USA). Cells were frozen in equal aliquots and stored in liquid nitrogen. One day before engraftment, the cells were thawed and cultured in Advanced RPMI 1640 Reduced Serum Medium (12633-012; ThermoFisher, Waltham, MA, USA) supplemented with 10% FBS and 2 mM L-Glutamine at a temperature of +37 °C, humidity over 90%, and a 5% CO_2_ atmosphere.

On the day of engraftment, to determine the density and viability of cells, as well as the initial proportions of hCD3^+^ and hCD4^+^ populations, the suspension sample was stained with solutions of fluorescent antibodies (Table 2) in accordance with the manufacturer’s recommendations. Immunophenotyping was performed using a NovoCyte 3000VYB flow cytometer (Agilent Technologies, Santa Clara, CA, USA).

To obtain the concentration required for administration, the cell suspension was diluted with cooled to +2 to +8 °C 1× DPBS. Immediately before injection, the diluted suspension was divided into 0.3 mL aliquots of the appropriate concentration, which were stored on ice for no more than 30 min.

To prepare the second type of graft, hPBMCs were cultured in RPMI1640 medium supplemented with 10% FBS and 2 mM L-Glutamine at a temperature of +37 °C, humidity over 90%, and a 5% CO_2_ atmosphere for 10 days. After 24 h, 375 IU/mL of interleukin-2 (11017; NPK BIOTECH, Saint Petersburg, Russia) and Dynabeads Human T-Activator CD3/CD28 (11132D; ThermoFisher, USA) were added to the cell suspension at a ratio of 1 bead per 1 cell. On days 3, 5, and 7 of cultivation, the cell suspension was 4-fold diluted with fresh RPMI1640 supplemented with 10% FBS, 2 mM L-Glutamine, and 375 IU/mL interleukin-2. Determination of hT-cell density and viability, as well as the initial proportions of hCD3^+^ and hCD4^+^ populations, was carried out in the same way as for hPBMCs.

Based on the results of cell density determination in the suspension, a volume containing the required number of hT-cells was aspirated. The cells were sedimented by centrifugation at 300× *g* and a temperature of +4 °C for 10 min. The supernatant was removed, and the sediment was resuspended in the appropriate volume of hPBMCs suspension. The hPBMCs suspension was prepared at the time of mixing according to the method described above. Immunophenotyping, division of the suspension into aliquots, and storage before injection were carried out similarly to hPBMCs.

### 2.3. Myeloablative Conditioning of Mice

To determine suitable irradiation conditions, we tested doses of 1.0 Gy, 1.5 Gy, and 2.0 Gy. And 1.0 Gy resulted in early recovery of the mice’s own immune system, which would have prevented full engraftment of HCs. In turn, 2.0 Gy turned out to be too high, since hemorrhages developed in the organs (especially in the brain) of the mice, and almost all of them were euthanized within 2 weeks after irradiation. Therefore, whole-body irradiation by orthovoltage X-rays with a dose of 1.5 Gy was considered sufficient to safely induce long-term myeloablation. The irradiation conditions were determined on the day of exposure based on the readings of the UNIDOS E electrometer (PTW Dosimetry, Freiburg, Germany).

All mice were irradiated in a CIX3 X-ray irradiator cabinet (Xstrahl LTD, Walsall, UK) one day prior to engraftment. Mice were previously anesthetized by inhalation of Isoflurane (Laboratorios Karizoo, Barcelona, Spain) using a Biosthesia 300 system (Vilber Lourmat, Collégien, France).

### 2.4. Administration of the Cells

Cell suspensions in a volume of 0.2 mL containing the appropriate number of cells were injected into the lateral tail vein according to Table 1. Control mice received 0.2 mL of 1× DPBS. Injections were performed using a syringe with a 30 G needle (1046152, Vogt Medical GmbH, Karlsruhe, Germany).

### 2.5. Determining the Effect of Engraftment on the Welfare of Mice

Mice were monitored daily for GVHD progression throughout the study. Mice were observed twice-weekly until humane endpoints were reached according to an adaptation of the methodology described by Seng, A. and Markiewicz, M.A. [16]:

- The mice were deprived of food and water. Their activity was monitored for 5 min. Scoring was as follows: 0 = mouse started moving within 2 min and continued moving; 1 = mouse took more than 2 min to get up and moved slowly; 2 = mouse did not get up within 5 min and moved only when touched;
- Weight loss: 0 = <10%, 1 = 10–20% and 2 = >20%;
- Mouse posture: 0 = no change, 1 = hunched posture at rest, 2 = hunched posture makes movement difficult;
- Fur texture: 0 = unchanged, 1 = light to moderate ruffle, 2 = heavy ruffle;
- Skin condition: 0 = no change, 1 = peeling in hairless areas, 2 = obvious peeling of the skin in areas of hair loss.

Humane endpoints were defined as follows:

- Assigning a mouse 7 or more scores;
- Body weight loss > 25%.

If humane endpoints were not achieved, the mice were euthanized at 82 d.p.i.

### 2.6. Blood Samples Collection

Whole blood samples for CBC and FACS were collected via inferior vena cava puncture at 5, 19, 26, 33, 40, 47, 54, 61, 68, 75, and 82 d.p.i. Blood samples for ELISA were collected before HCs engraftment and at the end of the study, at days −8 and 82, respectively.

For CBC and FACS, blood was collected in Microtainer K2E tubes (365975; Becton Dickinson, Franklin Lakes, NJ, USA), and for serum collection in MiniCollect tubes (450533; Greiner Bio-One, Kremsmünster, Austria).

### 2.7. Evaluation of Engraftment

The main factor of interaction between recipients and grafts was assessed by fluorescence-activated cell sorting (FACS) of mouse whole blood samples. For this purpose, we used a gating strategy that included separation of cell populations based on their forward (FSC) and side (SSC) scattering, isolation of a single cell population, and analysis of the expression of specific markers in a lymphocyte population separated by size:

- Determination of the lymphocyte fraction;
- Determination of the single lymphocyte fractions;
- Determination of the fraction of viable lymphocytes;
- Determination of the population of hT-cells (hCD3^+^mCD45^−^) and mouse lymphocytes (hCD3^−^mCD45^+^);
- Determination of the fraction of human T-helper cells (hT-helper cells) in the hT-cell population (Figure S1).

The antibody conjugates and dyes presented in Table 2 were used for FACS of mouse blood samples.

The degree of chimerism was calculated using the formula:

(1) $$Degree\;of\;chimerism=\frac{N{hCD3}^{+}}{N{hCD3}^{+}+N{mCD45}^{+}}\times 100\%,$$

N—number of cells.

As is known, donor hT-helper cells are directly responsible for the progression of GVHD [17]. In addition, HIV uses hCD4^+^-cells to reproduce and spread in the body. Therefore, we monitored their dynamics throughout the study.

The fraction of hT-helper cells in the T-cell population was calculated using the formula:

(2) $$\mathrm{hT-helper}\;cells\;fraction=\frac{N{hCD3}^{+}{hCD4}^{+}}{N{hCD3}^{+}{hCD4}^{+}+N{hCD3}^{+}{hCD4}^{-}}\times 100\%,$$

N—number of cells.

### 2.8. Evaluation of the Effect of Grafts on Mouse Hematopoiesis

To determine the effect of grafts on recipient hematopoiesis, we performed a complete blood count (CBC) of whole blood samples according to the following criteria: absolute white blood cell count (WBC), absolute red blood cell count (RBC), hemoglobin count (HGB), hematocrit (HCT), mean corpuscular volume (MCV), mean corpuscular hemoglobin (MCH), mean corpuscular hemoglobin concentration (MCHC), red cell distribution width (RDW), absolute platelet count (PLT), mean platelet volume (MPV), platelet distribution width (PDW), plateletcrit (PCT), absolute lymphocyte count (Lymph#), absolute monocyte count (Mon#), absolute granulocyte count (Gran#), relative lymphocyte count (Lymph%), relative monocyte count (Mon%), and relative granulocyte count (Gran%). CBC was performed by a Mindray BC-2800Vet automatic hematology analyzer (Mindray Bio-Medical Electronics Co., Ltd., Shenzhen, China).

In addition, we determined the dynamics of the concentration of mouse endogenous immunoglobulin G (mIgG) before the introduction of HCs and at the end of the study. For this purpose, the individual mouse serum samples were evaluated using the High-Sensitive ELISA Kit for Immunoglobulin G (HEA544Ra; Cloud-Clone Corp., Wuhan, China) according to the manufacturer’s instructions. Optical density was measured with a Multiskan FC plate photometer (Thermo Scientific, Waltham, MA, USA). The optical density results were processed using Skanlt RE 6.1 software (version 6.1.0.51, Thermo Scientific, Waltham, MA, USA).

### 2.9. Pathomorphology

During the necropsy, a macroscopic examination was performed, accompanied by measurement of the organ weight. The weight of each organ or organ complex was determined by an Ohaus Pioneer PX 223 scale (Ohaus, Parsippany, NJ, USA). The lungs and kidneys were weighed in pairs. The relative weight of organs was determined using the formula:

(3) $$Relative\;weight\;of\;organ=\frac{Organ\;weight}{Body\;weight}\times 100\%,$$

The spleen, lungs, kidneys with adrenal glands, liver, red bone marrow (BM) with sternum, small intestine with mesentery, brain, and uterus with ovaries were removed for histological studies.

### 2.10. Histology and Immunohistochemistry

Preparation of formalin-fixed and paraffin-embedded tissue sections was carried out according to routine procedure. Samples of organs and tissues were fixed in a 10% buffered formalin solution. They were then successively rehydrated with increasing concentrations of isopropyl alcohol and embedded in paraffin. Paraffin blocks were cut into 4–5 µm-thick sections by a Rotary 3003 rotary microtome (PFM medical, Cologne, Germany).

Hematoxylin and eosin (H&E) staining was performed according to the routine technique. The slides were evaluated at 100× and 400× magnification using an Optika B-500TPL microscope (Optika Microscopes, Bergamo, Italy) equipped with a photo/video recording system.

HCs in sections were determined by immunochromogenic detection of 3,3-diaminobenzidine tetrahydrochloride (DAB)-labeled anti-hCD4+ antibodies (DF16080; Affinity Bioscience, Zhenjiang, China). Polyperoxidase-anti-Rabbit IgG (E-IR-R215B, Elabscience, Wuhan, China) was used as a secondary antibody. DAB working solution was prepared by diluting DAB Concentrate (E-IR-R215D, Elabscience, China) in DAB Substrate (E-IR-R215E, Elabscience, China) according to the manufacturer’s recommendations.

### 2.11. Statistical Analysis

Statistical data processing was performed using the GraphPad Prism 9.0.0 software product. Survival analysis was performed using the Kaplan–Meier method in the modifications of Mantel–Cox and Gehan–Breslow–Wilcoxon. The dynamics of CBC indexes and the interaction of grafts and the recipient in vivo were examined by a two-way analysis of variance (ANOVA), determination of the Pearson linear correlation coefficient (*r*), Spearman’s rank correlation coefficient, and Tukey’s multiple comparisons test. Graphical presentations of the results of statistical analyses were constructed based on the mean values for each group of animals, including standard deviations (when applicable).

To determine *r*, we used the median values for the group of mice for the entire observation period, but *rs* was determined only for each type of graft. The interpretation of *r* and *rs* values is presented in Table 3. The threshold value of the significance level was set at 0.05. If the *p*-value was below the threshold value of the significance level, the result was considered statistically significant.

## 3. Results

### 3.1. Effects of the Type and Concentration of the Graft on the GVHD Progression

**Figure 1:**
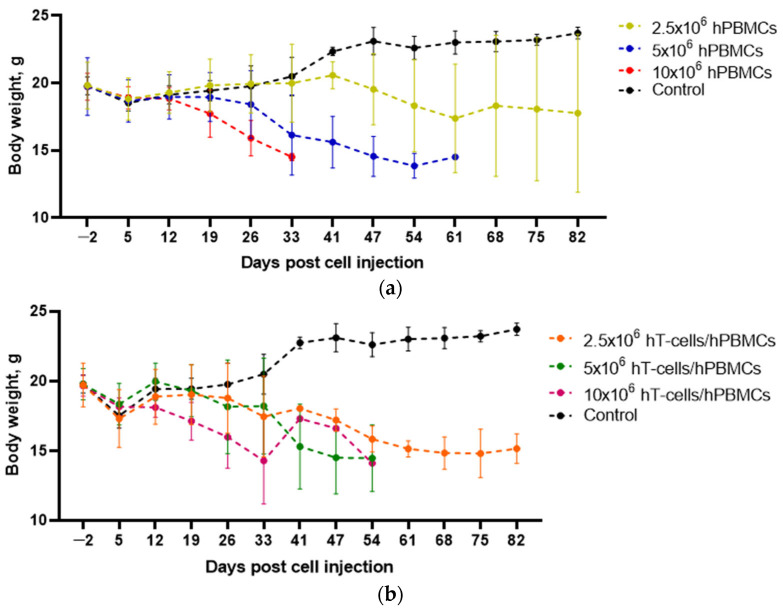
Effect of the type and concentration of the graft on body weight dynamics. (**a**) in 2.5 × 10^6^ hPBMCs mice, body weight loss began to be recorded at 47 d.p.i. and (**b**) in 2.5 × 10^6^ hT-cells/hPBMCs mice, body weight loss began to be observed at 19 d.p.i.

Although GVHD progression was assessed using several parameters, body weight was the leading measure of animal welfare. The greatest weight loss was observed in 5 × 10^6^ hPBMCs and 10 × 10^6^ hPBMCs mice starting at 12 d.p.i. (Figure 1a). A decrease in body weight was also observed in hT-cells/hPBMCs mice starting at 12 d.p.i. (Figure 1b).

In turn, ANOVA demonstrated the presence of a significant relationship between the concentration of both types of grafts and the dynamics of body weight over time (Table 4).

**Figure 2:**
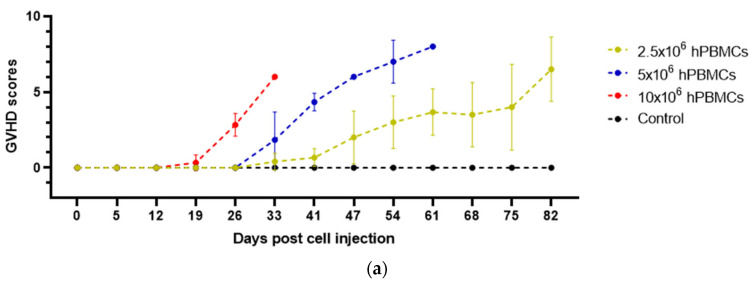

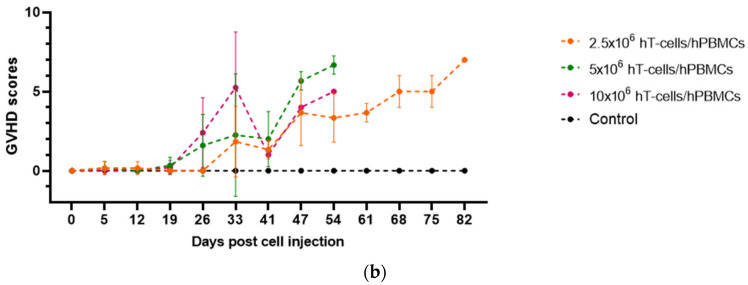
Effect of the type and concentration of the graft on the dynamics of GVHD. (**a**) With increasing concentration of injected hPBMCs, the time to onset of GVHD signs decreases, and (**b**) the progression of GVHD in hT-cells/hPBMCs mice was nonlinear.

The concentration of the grafts played a decisive role in the development of GVHD signs. Thus, in 10 × 10^6^ hPBMCs mice, the first signs began to be observed at 12 d.p.i., while in 5 × 10^6^ hPBMCs mice at 26 d.p.i. (Figure 2a). Signs of GVHD progressed in all hT-cells/hPBMCs groups throughout the study (Figure 2b).

ANOVA demonstrated an interaction between cell type and concentration and GVHD progression over time for all grafts (Table 5).

### 3.2. The Survival of Humanized Mice Depends on the Type and Concentration of the Graft

One mouse from each of the 2.5 × 10^6^ hPBMCs and 5 × 10^6^ hPBMCs groups died of non-specific causes. All 10 × 10^6^ hPBMCs mice, two 5 × 10^6^ hPBMCs mice, and one 2.5 × 10^6^ hPBMCs mouse were euthanized upon reaching humane endpoints. In the hT-cells/hPBMCs groups, five 5 × 10^6^ hT-cells/hPBMCs mice and 10 × 10^6^ hT-cells/hPBMCs mice and one 2.5 × 10^6^ hT-cells/hPBMCs mouse were euthanized upon reaching humane endpoints.

**Figure 3:**
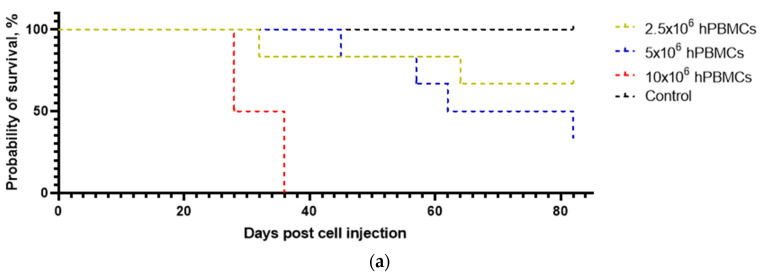

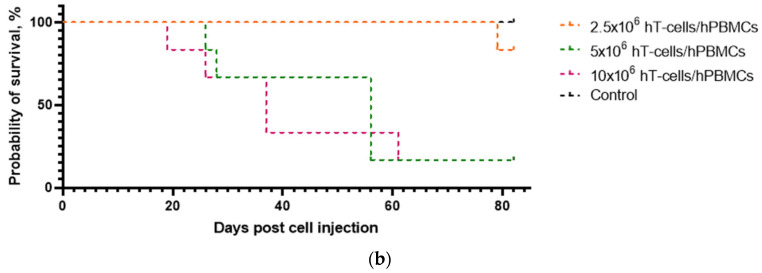
Effect of the type and concentration of the graft on survival probability. (**a**) 10 × 10^6^ hPBMCs mice were excluded from the study for ethical reasons, and (**b**) engraftment of 2.5 × 10^6^ hT-cells/hPBMCs had the least negative impact on the survival of mice.

The survival curves of hPBMC and hT-cell/hPBMC mice showed significant differences between each other (Figure 3).

Survival analysis determined the effect of the concentration of both types of grafts on the survival probability of mice (Table 6). A mean survival of 72% was determined for 5 × 10^6^ hPBMCs mice, 32% for 10 × 10^6^ hPBMCs mice, 56% for 5 × 10^6^ hT-cells/hPBMCs mice, and 37% for 10 × 10^6^ hT-cells/hPBMCs mice.

### 3.3. Analysis of the Dynamics of CBC Indices

Given the survival analysis results, statistical analysis was performed only for 2.5 × 10^6^ hPBMCs and 2.5 × 10^6^ hT-cells/hPBMCs mice. However, for clarity, we illustrated the dynamics of graft indices for all concentrations.

#### 3.3.1. Leukocyte Indices

Moderate leukocytosis was observed in mice of the experimental groups throughout almost the entire study (Figure S2a,b). Peak WBC at 19 d.p.i. is associated with regenerative processes in the hematopoietic organs after myeloablation. At the same time, relative stabilization of WBC in 2.5 × 10^6^ hPBMCs mice occurred at 54 d.p.i., and in 2.5 × 10^6^ hT-cells/hPBMCs mice at 61 d.p.i. At the same time, no statistically significant relationship between the types of grafts and the dynamics of WBC was confirmed.

A similar pattern was observed for Lymph% and Gran% (Figure S2c,d,g,h). Relative granulocytopenia was observed throughout the study in all experimental groups. However, absolute granulocytosis was noted only in hT-cells/hPBMC mice (Figure S2n). Moderate absolute lymphocytosis was also observed in all groups, but it was more pronounced in mice that received the minimum number of grafts of both types (Figure S2i,j). Marked absolute monocytosis was noted from 26 to 61 d.p.i. only in hT-cells/hPBMCs mice (Figure S2l).

For all groups, no significant relationship was found between the types of grafts and Lymph# and Lymph%, as well as Gran%. In addition, the effect of 2.5 × 10^6^ hPBMCs on the dynamics of Gran#, as well as 2.5 × 10^6^ hT-cells/hPBMCs on the dynamics of Mon#, was not confirmed.

#### 3.3.2. Erythrocyte Indices

Despite some variability between groups, the dynamics of RBC showed a general tendency to gradually decrease, starting from the first days of observation (Figure S3a,b). At the same time, RBC did not depend on the engrafted hPBMCs, but had a statistically significant relationship with the concentration of hT-cells/hPBMCs.

Since HGB is directly dependent on RBC, their dynamics graphs are practically identical (Figure S3c,d). However, a statistically significant relationship between HGB and the types of graft was confirmed for all groups. Despite the fact that the curves of HCT and RBC dynamics are also very similar, the analysis of the dynamics did not reveal a statistically significant relationship for hPBMCs, but confirmed it for hT-cells/hPBMCs.

Although MCV is an estimated parameter, the effect of both types of grafts on its dynamics was established. MCH is also an estimated parameter, but the effect of the type of graft on its dynamics was not confirmed for either group. For MCHC dynamics, an interaction with the type of graft was also confirmed. The increase in RDW was also due to the introduction of grafts, and for all concentrations (Figure S3m,n).

#### 3.3.3. Platelet Indices

The dynamics of PLT had an inverse dose-dependent effect on the graft concentration (Figure S4a,b). Thus, the maximum PLT was observed in mice that received the minimum of HCs. At the same time, the lowest PLT was recorded in mice that received grafts at the maximum concentration. At the same time, a statistically significant relationship was established between the graft concentration and the dynamics of PLT.

The group dynamics of MPV showed an inverse trend to PLT, indicating a direct and significant effect of graft concentration on thrombocytopoiesis (Figure S4c,d). The effect of the type and concentration of the HCs on PDW was not established. However, an inverse dose-dependent effect on PCT was determined for both types of grafts.

### 3.4. The Type and Concentration of the Graft Do Not Significantly Activate B-Cell Immunity in Mice

**Figure 4:**
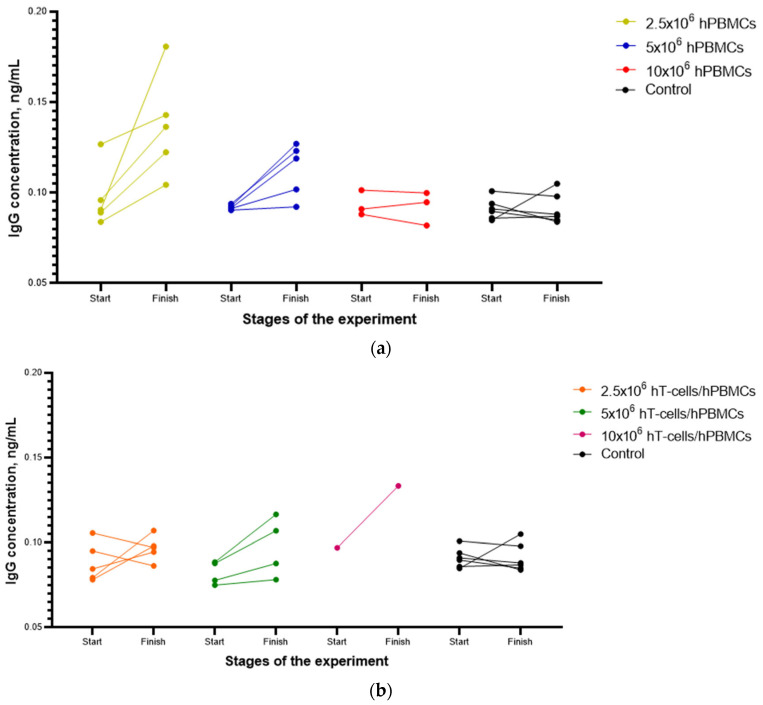
Individual effects of the type and concentration of the graft on immune activation in mice. (**a**) A slight increase in mIgG concentration was observed in one mouse of 2.5 × 10^6^ hPBMCs and (**b**) a slight increase in mIgG concentration was recorded in one mouse of 10 × 10^6^ hT-cells/hPBMCs. Some data are missing due to mouse death.

Analysis of the effect of the type of graft on the activation of B-cell immunity in mice demonstrated insignificant fluctuations in mIgG concentration, both upward and downward, in all groups (Figure 4). Thus, the effects of preliminary myeloablation and the type of graft on the stimulation of B-cell formation in mice over time were insignificant.

ANOVA demonstrated a statistically significant relationship between the initial and final mIgG concentrations. Tukey’s multiple comparison test indicated that the highest probability of stimulating B-cell production was achieved when 2.5 × 10^6^ hPBMCs were used as a graft.

### 3.5. The Type of Graft Determines the Dynamics of HC Engraftment but Not the Dynamics of hT-Helper Cells Concentration After Engraftment

**Figure 5:**
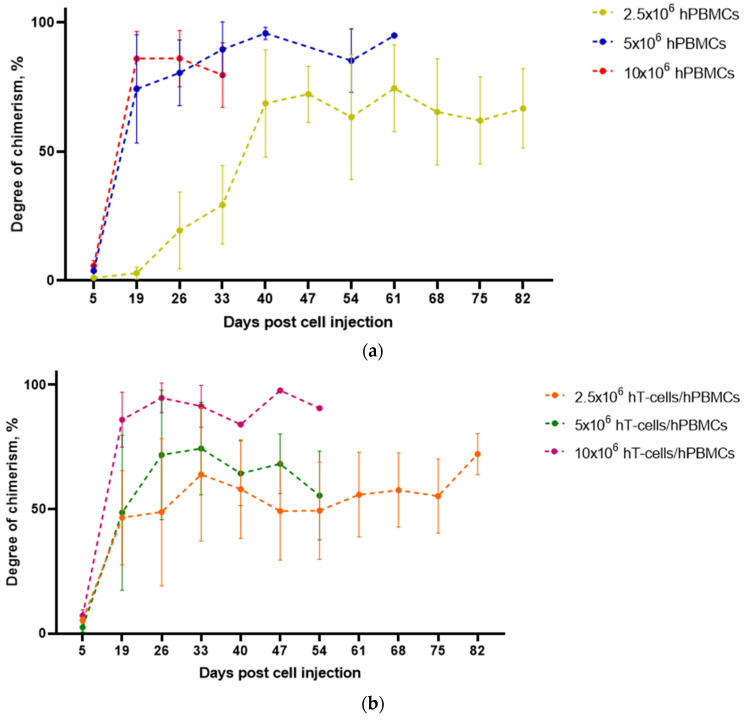
Dynamics of the degree of chimerism. (**a**) 5 × 10^6^ hPBMCs and 10 × 10^6^ hPBMCs mice showed a burst of degree of chimerism at 19 d.p.i. and (**b**) degree of chimerism in 2.5 × 10^6^ hT-cells/hPBMCs mice was more stable than in other mice.

The interaction of grafts and recipients was assessed based on the dynamics of changes in the proportion of hCD3^+^ T-cells relative to the mCD45^+^ T-cell population in whole blood samples. In hPBMCs and hT-cells/hPBMCs mice, a burst of HC populations was observed from 5 to 19 d.p.i. After this, in the 10 × 10^6^ hPBMCs mice, their decrease began, which lasted until the mice reached humane endpoints. In the remaining groups, the dynamics of the HCs’ number had a wave-like character (Figure 5).

The highest number of HCs was observed in 10 × 10^6^ hT-cells/hPBMCs mice, reaching 99% at 47 d.p.i. However, already at 54 d.p.i., the remaining mice in this group reached humane endpoints. In turn, the maximum degree of chimerism of 2.5 × 10^6^ hPBMCs mice was ~80% at 61 d.p.i., and the mean value for the entire observation period was more than 48%. At the same time, the maximum degree of chimerism of 2.5 × 10^6^ hT-cells/hPBMCs mice was ~72% at 82 d.p.i., and the mean value for the entire study was more than 47%. ANOVA demonstrated a statistically significant relationship between the types of grafts and engraftment dynamics over time.

An increase in the fraction of hT-helper cells in both hPBMCs and hT-cells/hPBMCs mice was observed up to 33–54 d.p.i. (Table S1).

**Figure 6:**
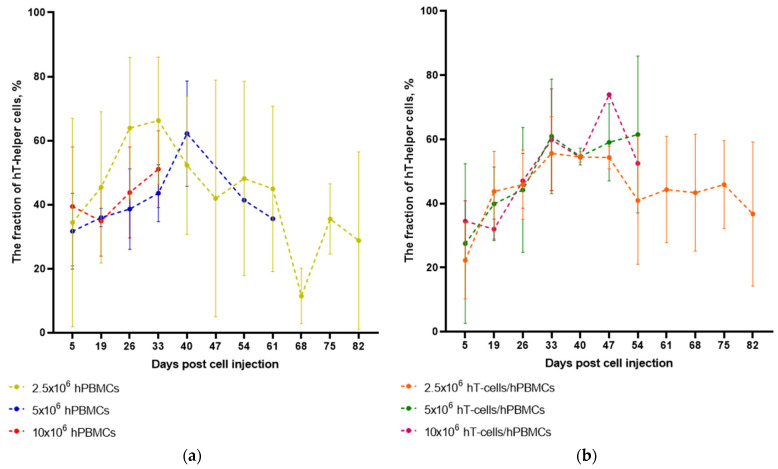
Dynamics of the hT-helper cells. (**a**) Administration of 10 × 10^6^ hPBMCs results in a transient decrease in the fraction of hT-helper cells during 21 d.p.i. and (**b**) administration of 2.5 × 10^6^ hT-cells/hPBMCs results in the long-term maintenance of a constant concentration of hT-helper cells.

At the same time, at 19 d.p.i., mice that received the maximum of HCs showed a slight decrease, followed by an increase in the fraction of hT-helper cells (Figure 6). ANOVA did not confirm the relationship between the type of graft and the dynamics of the fraction of hT-helper cells in the T-cell population over time.

### 3.6. Definition of Linear Correlations

The magnitude of the linear correlation between the mean degree of chimerism and GVHD progression was determined using *r* (Table 7). The highest *r* value was observed in 2.5 × 10^6^ hPBMCs mice. However, the correlation was also positive in the remaining mice.

Since the development of GVHD is directly dependent on the activity of hT-helper cells, we also determined the degree of their correlation (Table 8).

The obtained data demonstrated the presence of a low positive correlation in 5 × 10^6^ hPBMCs and 2.5 × 10^6^ hT-cells/hPBMCs mice. A strong positive correlation was observed in 10 × 10^6^ hPBMCs, 5 × 10^6^ hT-cells/hPBMCs, and 10 × 10^6^ hT-cells/hPBMCs mice. And only in 2.5 × 10^6^ hPBMCs mice, a moderate negative correlation was noted.

In the same way, we determined the possible correlation between the GVHD score and the count of blood cell populations. In the case of WBC, it turned out that a positive correlation is possible only with 10 × 10^6^ hPBMCs (Table 9). Regarding RBC and PLT, a pronounced negative correlation was observed for all types of grafts. The exception was mice that received a moderate number of grafts. In the case of PLT, they showed a low negative correlation. As for the possible correlation between Lymph# and the GVHD score, both negative and positive low and moderate correlations were observed. Only for 10 × 10^6^ hPBMCs, a strong positive correlation was determined.

Finally, the presence of a possible relationship between the degree of chimerism and the count of blood cell populations was checked. As in the case of GVHD, the correlation between WBC and the degree of chimerism was positive and quite pronounced in all mice. The correlation between the degree of chimerism with RBC and PLT was again negative and quite pronounced (Table 10). As for Lymph#, the correlation was positive for all groups.

### 3.7. Macromorphological Analysis

The most common pathologies detected during necropsy were anemia and atrophy of organs and tissues (Table 11).

For postmortem assessment of the effect of GVHD on the body of mice, the liver, spleen, kidneys, lungs, brain, and uterus with ovaries were identified as target organs. These organs are the most accessible for the most accurate determination of weight, since they can be removed without contamination with fragments of extrinsic tissue. In addition, we assumed the accumulation of HCs, including in these organs. Therefore, determining their relative weight may provide additional information for understanding the effect of grafts.

**Figure 7:**
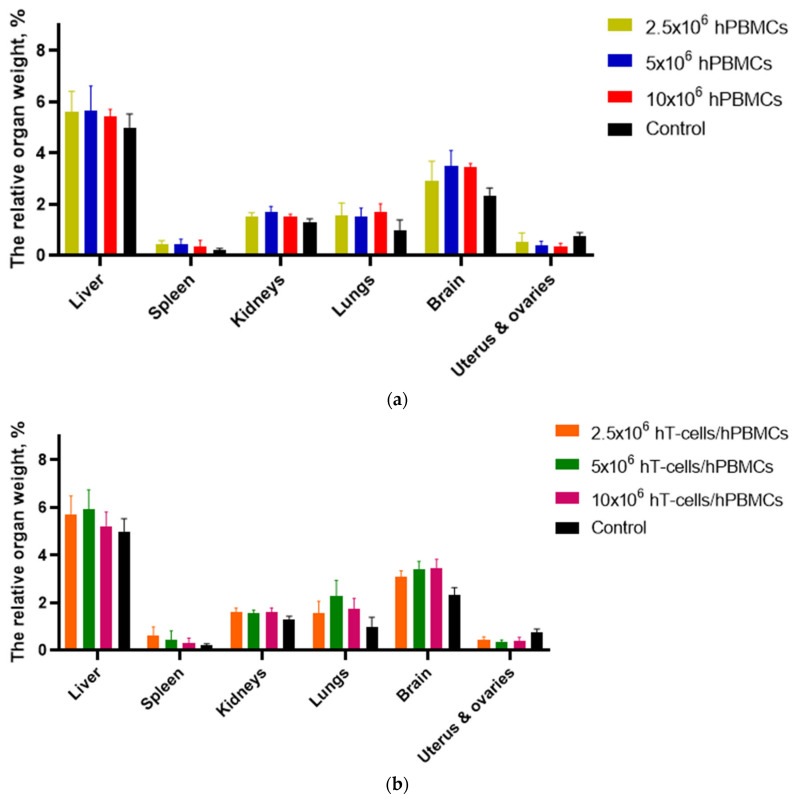
Target organ weights relative to mouse body weight. (**a**) Uterine atrophy was the most prominent pathology in hPBMCs mice, and (**b**) the most severe liver and spleen atrophy was observed in 5 × 10^6^ hT-cells/hPBMCs mice.

Since hPBMCs and hT-cells/hPBMCs mice showed a decrease in total body weight in association with the development of GVHD, a decrease in organ weight was also expected. However, Figure 7 shows the opposite trend. The relative weight of target organs in mice of the experimental groups, in most cases, exceeded the weight of control mice. The exception was the reproductive tract organs. This is explained by a decrease in appetite in mice with the development of GVHD, and as a consequence, a decrease in the volume of gastrointestinal tract contents. This, in turn, led to a decrease in the volume of adipose and muscle tissue. ANOVA of target organ relative weight values demonstrated the presence of a statistically significant relationship both between mice that received a graft of the same type in different concentrations and relative to control mice.

### 3.8. Micromorphological Examination

Given that the endpoints for euthanasia were reached unevenly, even within one experimental group, the pathological picture varied somewhat. Of particular interest was the effect of the type and concentration of the graft, both on the distribution of HCs and on the stimulation of the development of pathological features in organs and tissues. For this purpose, an analysis of H&E-stained tissue sections was performed.

**Figure 8:**
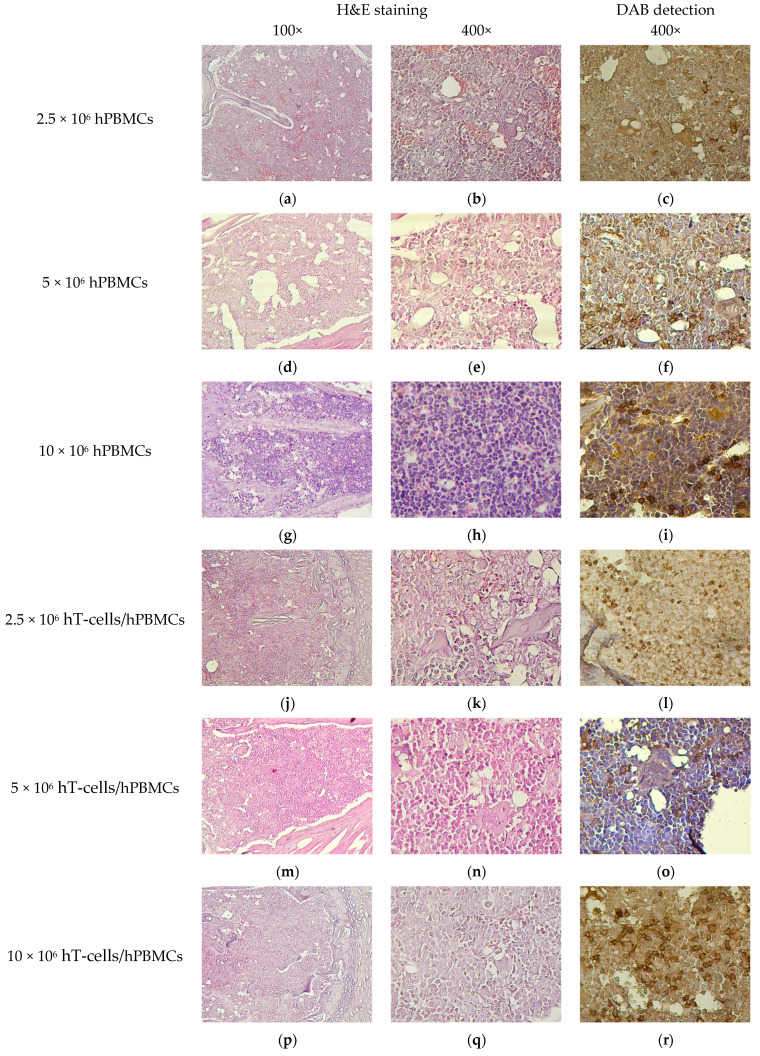

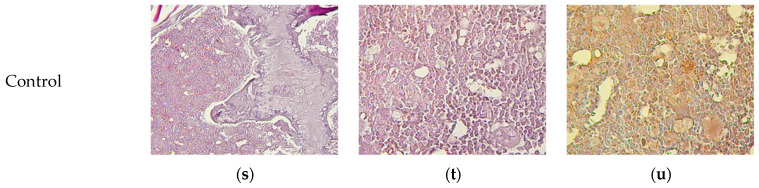
BM micromorphology. (**a**,**b**,**d**,**e**,**g**,**h**,**j**,**k**,**m**,**n**,**p**,**q**,**s**,**t**) H&E staining; (**c**) single scattered hCD4^+^-cells; (**f**,**i**) small clusters of hCD4^+^-cells; (**l**) moderate number of scattered hCD4^+^-cells; (**o**,**r**) extensive clusters of hCD4^+^-cells; and (**u**) no exogenous cells.

BM of mice that received the minimum of HC, regardless of the type of the graft, had moderate cytosis, expressed in numerous granulocytic and erythroid cells, as well as a moderate number of megakaryocytes. This corresponded to the picture observed in the control mice. At the same time, lower cytosis was observed in the BM of mice that received the maximum number of HCs, due to the replacement of normal BM components with connective tissue and fibrin. Single megakaryocytes were present, and the number of granulocytic and erythroid cells was low (Figure 8g,h,p,q). Moreover, the most pronounced atrophy was observed in 10 × 10^6^ hT-cells/hPBMCs mice (Figure 8p,q).

**Figure 9:**
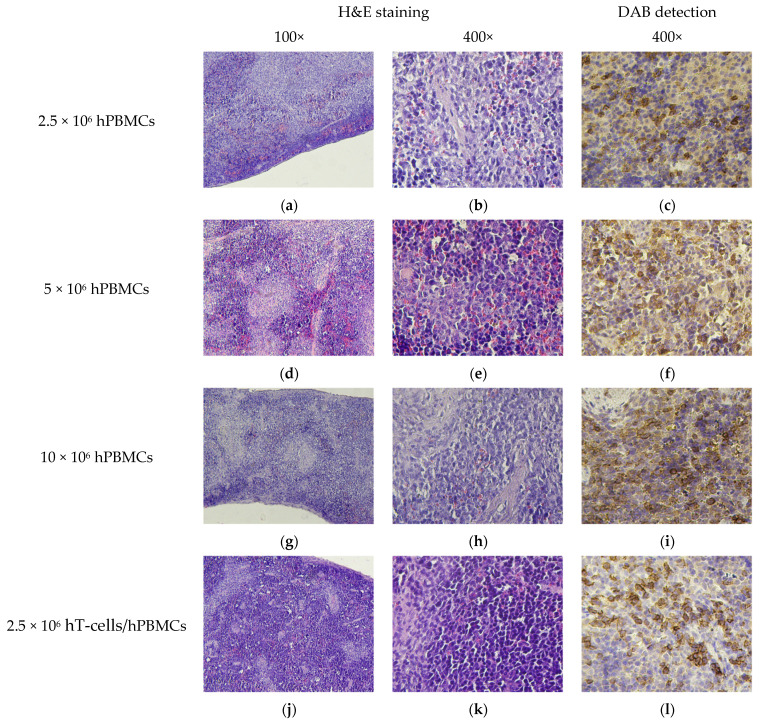

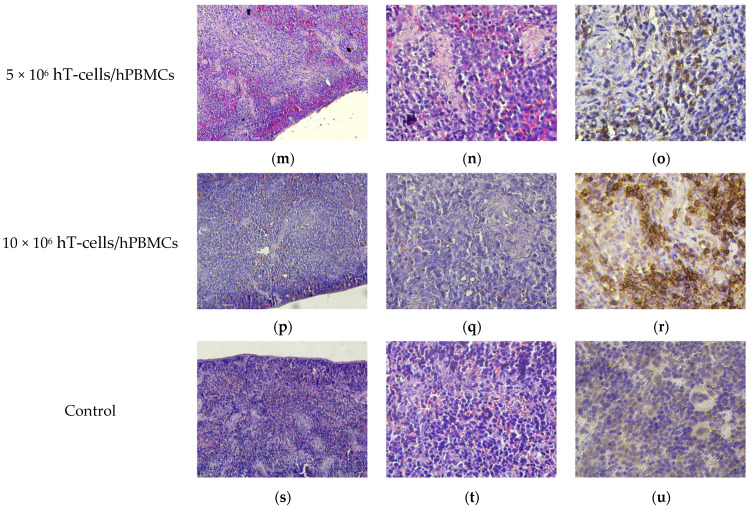
Micromorphology of the spleen. (**a**,**b**,**d**,**e**,**g**,**h**,**j**,**k**,**m**,**n**,**p**,**q**,**s**,**t**) H&E staining; (**c**) poor diffuse infiltration of hCD4^+^-cells; (**f**,**i**) moderate diffuse infiltration of hCD4^+^-cells; (**l**,**o**) small clusters of hCD4^+^-cells; (**r**) extensive clusters of hCD4^+^-cells; and (**u**) no exogenous cells.

Micromorphological features of the spleen also demonstrated the dependence of the occurrence and development of severe pathology on the type of graft. In 2.5 × 10^6^ hPBMCs mice, moderate cytosis and signs of extramedullary hematopoiesis were observed, indicating general anemia developing against the background of GVHD (Figure 9a,b). However, in 2.5 × 10^6^ hT-cells/hPBMCs mice, slight atrophy was developed, as well as moderate mononuclear infiltration of the white pulp. At the same time, in mice that received the maximum of HCs, pronounced parenchymal atrophy developed, accompanied by fibrosis of varying severity (Figure 9g,h,p,q). In addition, diffuse infiltrates of hCD4^+^-cells of varying cytosis were found in the white and red pulp.

**Figure 10:**
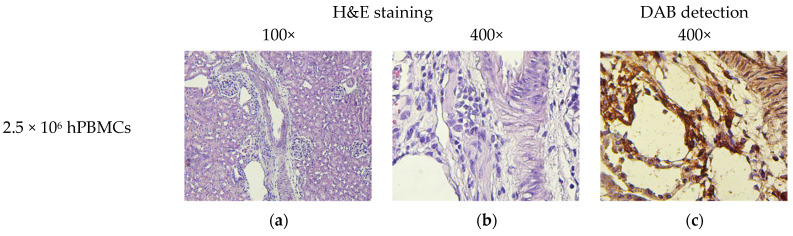

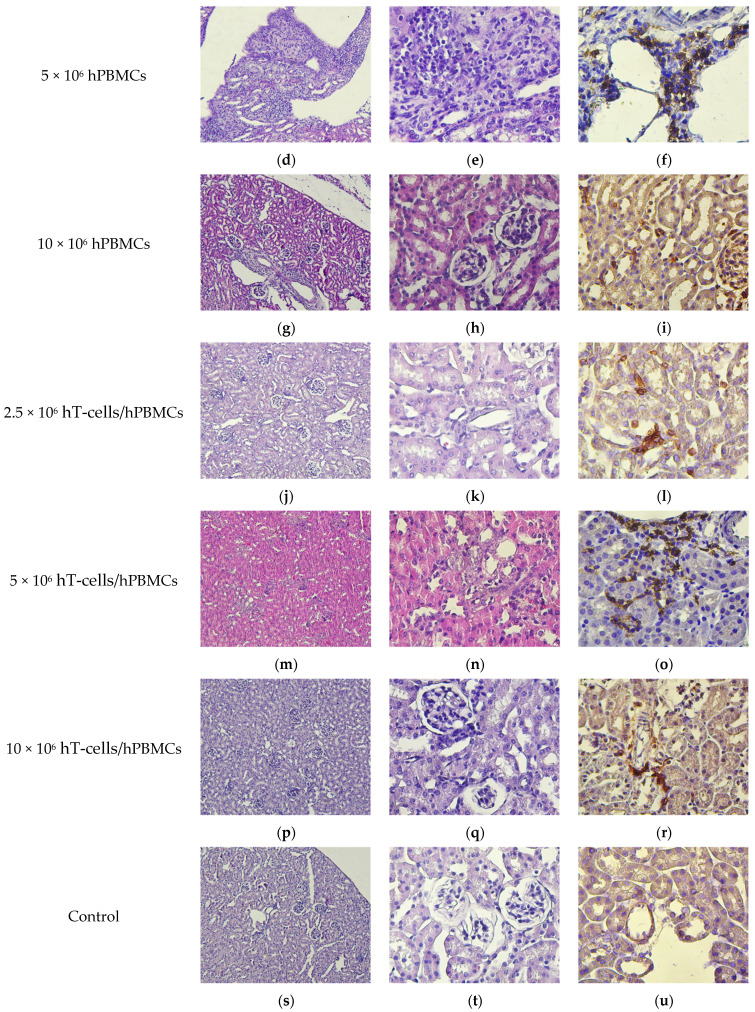
Micromorphology of the kidneys. (**a**,**b**) Moderate infiltration of mononuclear cells; (**c**) moderate infiltration of hCD4^+^-cells; (**d**,**e**,**m**,**n**) high infiltration of mononuclear cells; (**f**,**o**) high clusters of hCD4^+^-cells; (**g**,**h**) moderate diffuse infiltration of mononuclear cells; (**i**) moderate diffuse infiltration of hCD4^+^-cells; (**j**,**k**,**p**,**q**) small clusters of mononuclear cells; (**l**,**r**) small clusters of hCD4^+^-cells; and (**s**–**u**) exogenous cells are absent.

The morphological features of the kidneys also varied depending on the type of graft. The largest number of hCD4^+^-cells was detected near the vascular lumens (Figure 10).

**Figure 11:**
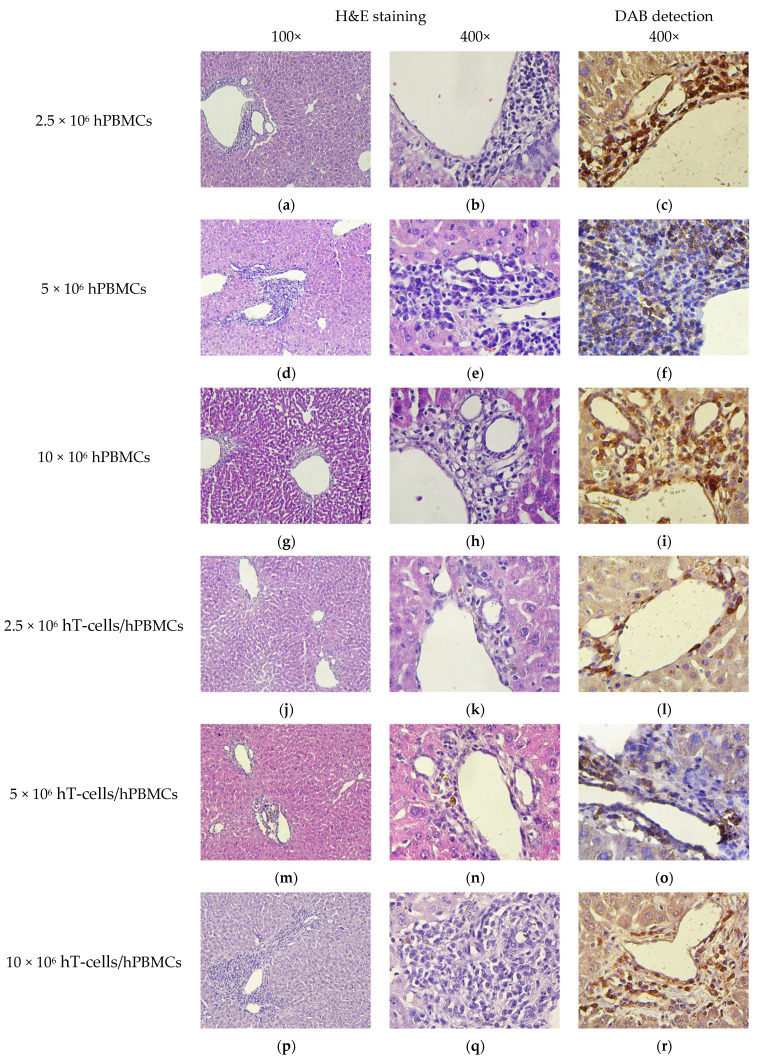

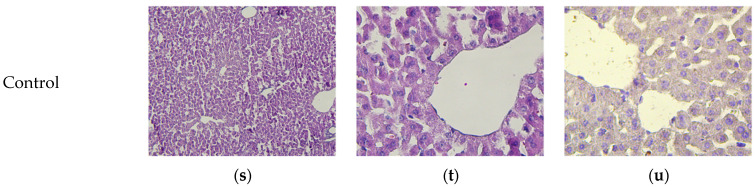
Micromorphology of the liver. (**a**,**b**) High diffuse infiltration of mononuclear cells; (**c**) high diffuse infiltration of hCD4^+^-cells; (**d**,**e**) high infiltration of mononuclear cells; (**f**) high infiltration of hCD4^+^-cells (**g**,**h**,**p**,**q**) moderate diffuse infiltration of mononuclear cells; (**i**,**r**) moderate diffuse infiltration of hCD4^+^-cells; (**j**,**k**,**m**,**n**) small clusters of mononuclear cells; (**l**,**o**) small clusters of hCD4^+^-cells; and (**s**–**u**) no exogenous cells.

Infiltrates of hCD4^+^-cells of various cytosis were also detected in the perivascular interstitium of the liver parenchyma of all humanized mice (Figure 11). In addition, moderate amyloidosis in 10 × 10^6^ hPBMCs mice and hemosiderosis of the perivascular interstitium in hT-cells/hPBMCs mice were detected.

**Figure 12:**
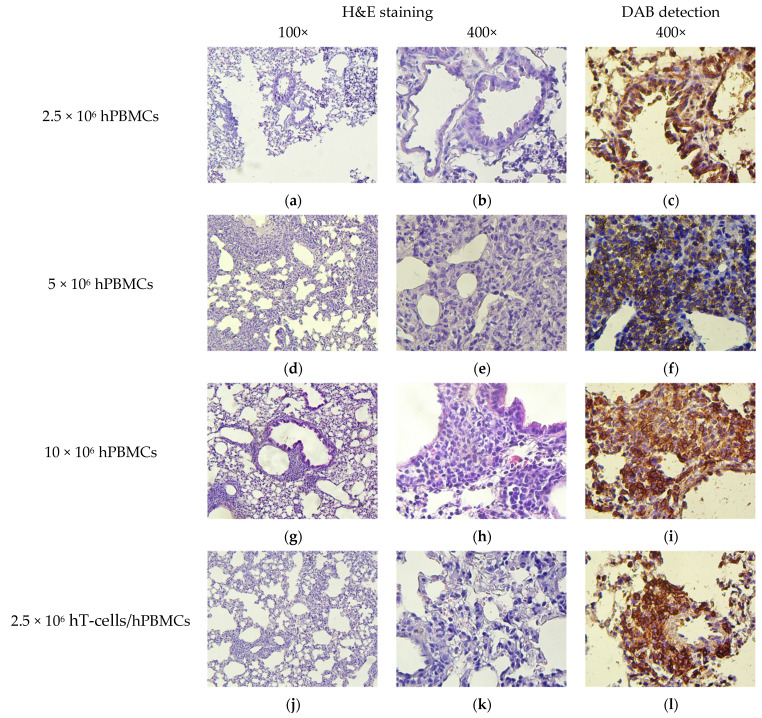

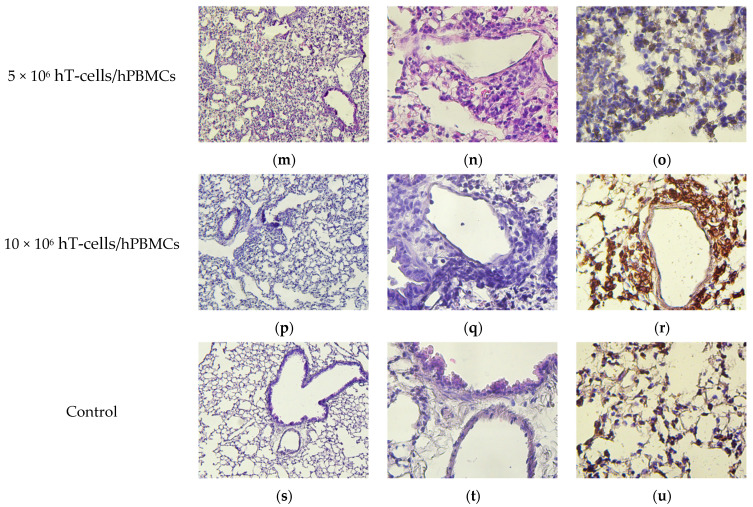
Micromorphology of the lungs. (**a**,**b**) Low diffuse infiltration of mononuclear cells; (**c**) low diffuse infiltration of hCD4^+^-cells; (**d**,**e**,**g**,**h**,**j**,**k**) extensive clusters of mononuclear cells; (**f**,**i**,**l**) extensive clusters of hCD4^+^-cells; (**m**,**n**,**p**,**q**) moderate clusters of mononuclear cells; (**o**,**r**) moderate clusters of hCD4^+^-cells; and (**s**–**u**) exogenous cells are absent.

The microscopic picture of the lungs in terms of relative infiltration of mononuclear cells resembled the features of the liver. Infiltrates were found in both the perivascular and peribronchial interstitium. Visually, the number of HCs in the perivascular interstitium was significantly higher than in the peribronchial (Figure 12).

**Figure 13:**
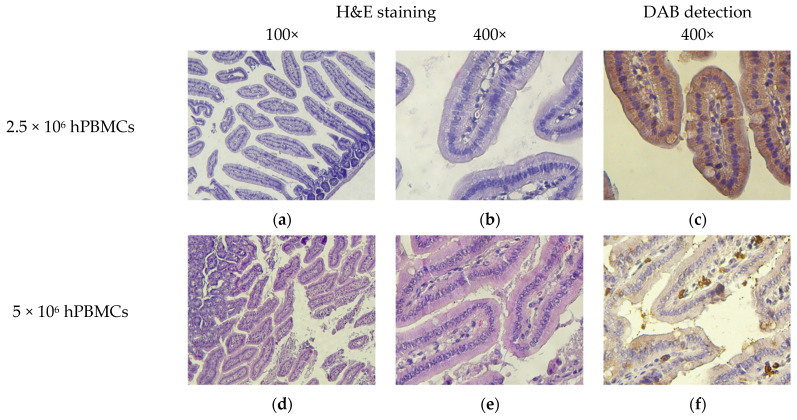

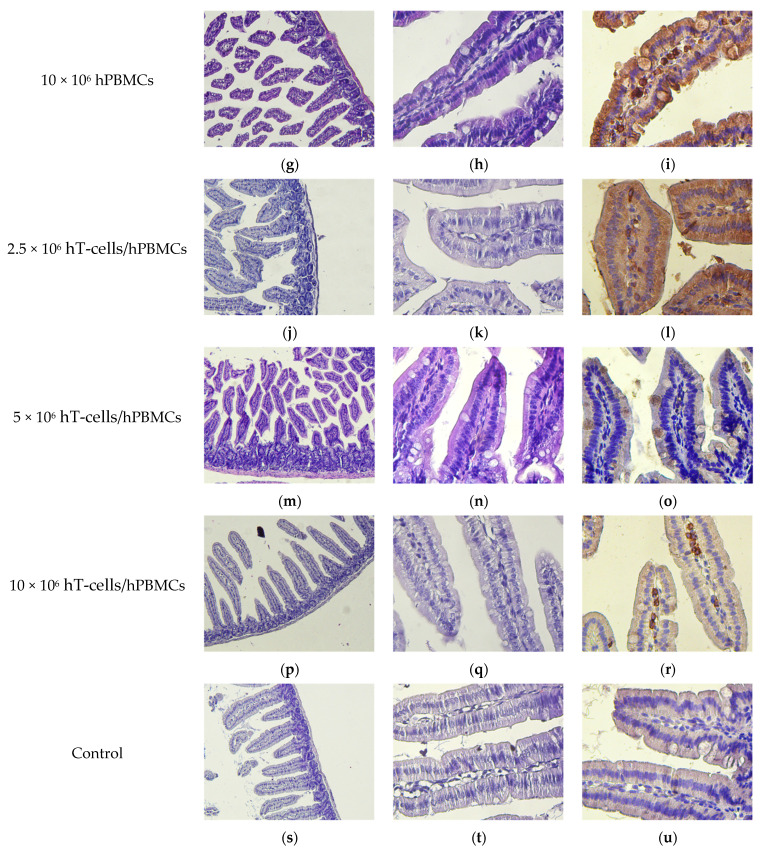
Micromorphology of the small intestine. (**a**,**b**) Single mononuclear cells; (**c**) single hCD4^+^-cells; (**d**,**e**,**g**,**h**,**j**,**k**) moderate clusters of mononuclear cells; (**f**,**i**,**l**) moderate clusters of hCD4^+^-cells; (**m**,**n**,**p**,**q**) small clusters of mononuclear cells; (**o**,**r**) small clusters of hCD4^+^-cells; and (**s**–**u**) no exogenous cells.

Although H&E-stained small intestinal sections showed no signs of HCs, immunochromogenic detection revealed the presence of hCD4^+^-cells in the lamina propria (Figure 13). In addition, hCD4^+^-cells were detected in the mesenteric lymph nodes.

**Figure 14:**
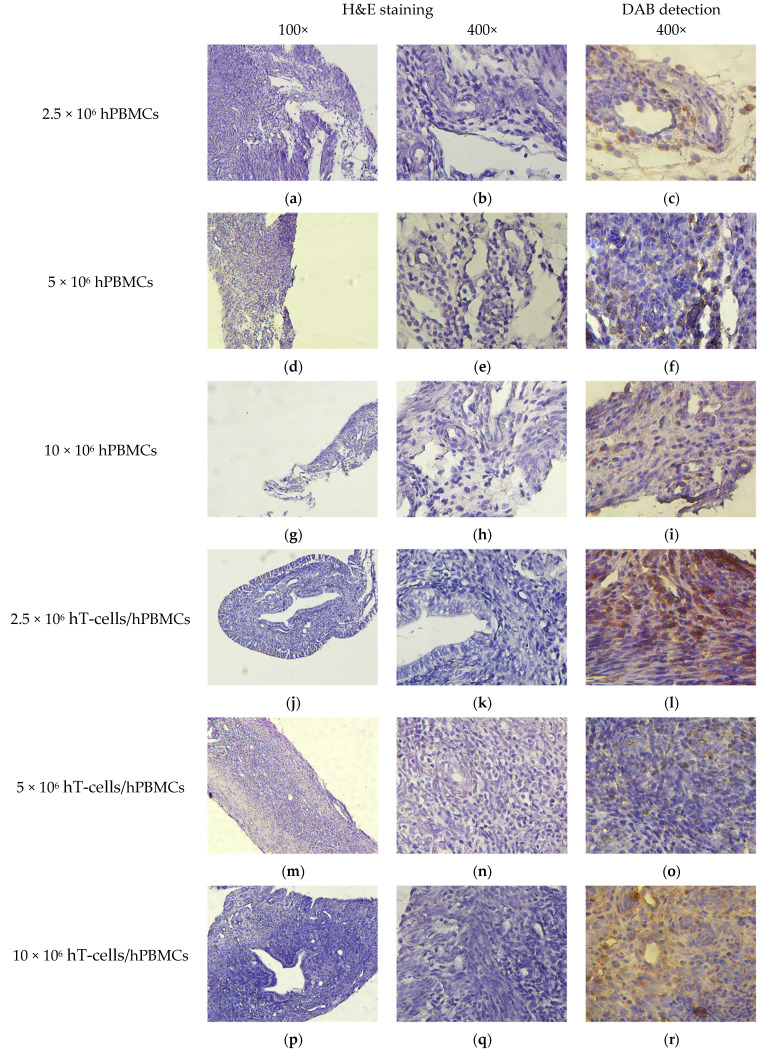

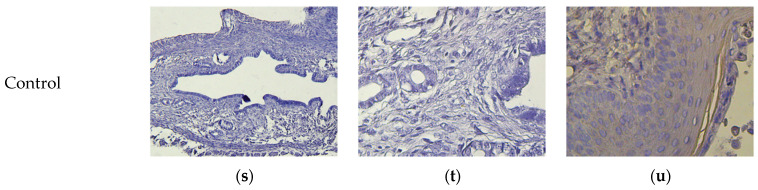
Micromorphology of the uterus. (**a**,**b**,**d**,**e**,**g**,**h**,**p**,**q**) Single scattered mononuclear cells; (**c**,**f**,**i**,**r**) single scattered hCD4^+^-cells; (**j**,**k**,**m**,**n**) moderate diffuse infiltration of mononuclear cells; (**l**,**o**) moderate diffuse infiltration of hCD4^+^-cells; and (**s**–**u**) exogenous cells are absent.

The degree of endometrial atrophy was not related to the graft concentration. Infiltrates of hCD4^+^-cells were detected in the myometrium as well as in the adjacent adipose tissue (Figure 14).

**Figure 15:**
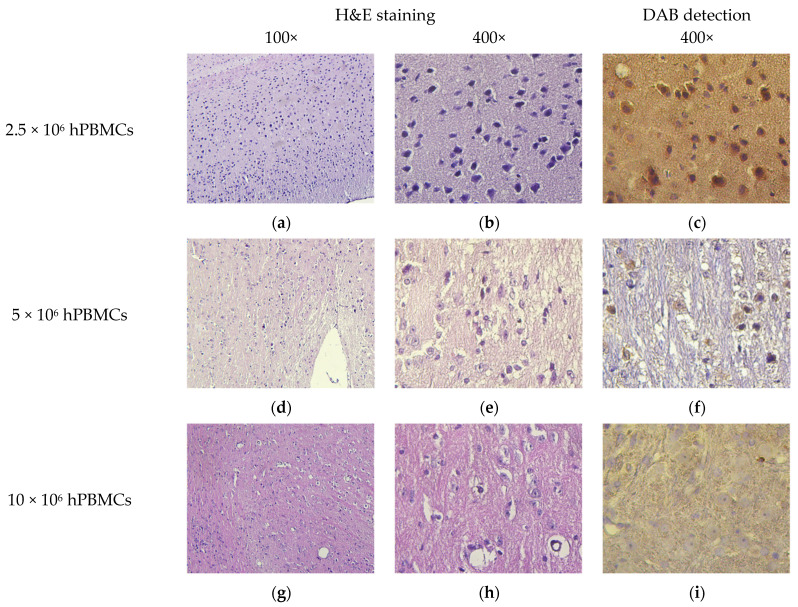

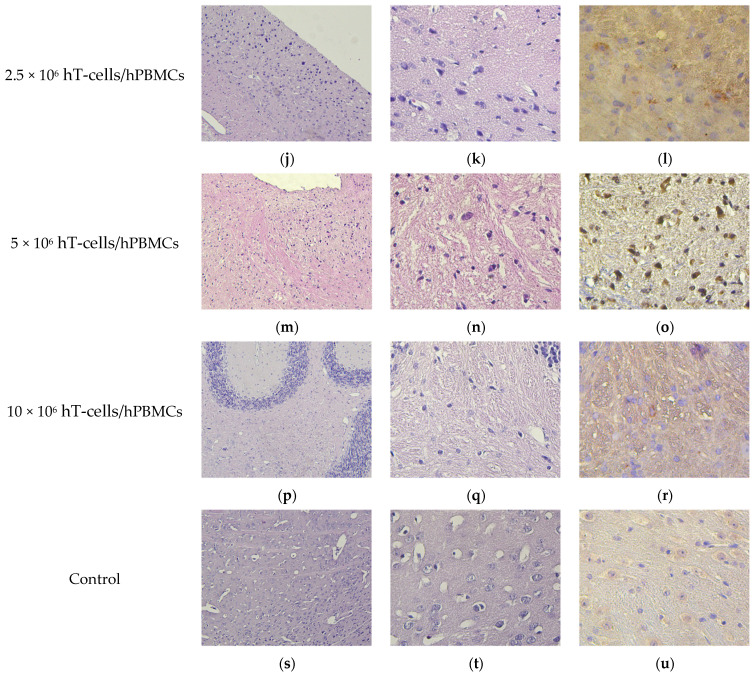
Micromorphology of the brain. (**a**,**b**) Moderate diffuse infiltration of mononuclear cells; (**c**) moderate diffuse infiltration of hCD4^+^-cells; (**d**,**e**,**g**,**h**,**j**,**k**,**m**,**n**,**p**,**q**) single scattered mononuclear cells; (**f**,**i**,**l**,**o**,**r**) single scattered hCD4^+^-cells; and (**s**–**u**) no exogenous cells.

When evaluating H&E-stained brain sections, our attention was focused on identifying obvious changes in the localization of HCs. In 2.5 × 10^6^ hPBMCs mice, a diffuse infiltration of mononuclear cells was detected in the external pyramidal cell layer of the cerebral cortex (Figure 15b). Immunochromogenic detection of hCD4^+^ confirmed the human origin of these cells. In the remaining mice, very few HCs were detected. It is noteworthy that hCD4^+^-cells were detected in the white matter of the cerebellum of 10 × 10^6^ hT-cells/hPBMCs mice (Figure 15l).

We determined the correlation between the number of hCD4^+^-cells in tissue sections and the type of the graft using three concentrations of each. No correlations were found for HCs localized in the liver of hPBMCs mice or in the small intestine and brain of hT-cells/hPBMCs mice (Table 12).

## 4. Discussion

C-NKG mice are commonly used as models for studying carcinogenesis or developing antitumor therapy, but we have found no reports of human immune system reconstitution in these mice [18,19]. However, there are a number of similar studies using mice with a similar genotype (Table 13). Thus, intravenous administration of 1.2 × 10^5^ hT-cells to preliminary irradiated 4- to 7-week-old female NOG (NOD.Cg-*Prkdc^scid^* Il2^rgtm1Sug^/JicTac) mice resulted in 10–30% hT-cell chimerism at 70–84 d.p.i. The fraction of hCD45^+^-cells reached (depending on the donor) approximately 90% and was recorded for 105 days. The number of hCD3^+^ T-cells did not exceed ~30–40% [12]. This information is somewhat different from the results we obtained. However, given the dynamics of the HCs in the blood of mice observed in our study, one can, with caution, predict similar results engrafting 2.5 × 10^6^ hPBMCs or 2.5 × 10^6^ hT-cells/hPBMCs. In addition, assessing the further prospects of the model in the study of HIV infection, such a duration of observation may not be necessary. Therefore, a concentration of 5 × 10^6^ hPBMCs may also be quite suitable.

Administration of large amounts of HCs not only induces pronounced chimerism but also stimulates the early development of GVHD. In our study, HCs were detected in the blood of 2.5 × 10^6^ hPBMCs and 2.5 × 10^6^ hT-cells/hPBMCs mice up to 82 d.p.i. However, given the dynamics of the chimerism level, the mean survival, and the dynamics of GVHD development, a longer observation period is acceptable for these mice. At the same time, among the other groups of mice for which it was possible to determine the mean survival, only 5 × 10^6^ hPBMCs mice could be distinguished, for which it was 72%.

In our study, 2.5 × 10^6^ hPBMCs mice achieved a mean peak hT-helper cells value (~73%) at 33 d.p.i., as did 2.5 × 10^6^ hT-cells/hPBMCs mice, which achieved ~55%. The mean peak hT-helper cells value in 5 × 10^6^ hPBMCs mice was recorded at 40 d.p.i. and was ~61%. These results look promising given the relatively short “observation windows” in the experiments of Lockridge J.L. et al. [21], Morillon Y.M. et al. [23], and Ali N. et al. [24] (Table 13). In all studies describing hPBMCs humanization of NSG mice (Table 13), the injected graft concentration was 10 × 10^6^ cells/mouse. In turn, we achieved impressive levels of chimerism using lower initial concentrations of hPBMCs.

We have determined that there was a statistically significant relationship between initial and final mIgG concentrations in 2.5 × 10^6^ hPBMCs mice, while the effects of preliminary myeloablation and the type of graft on B-cell stimulation were selective and insignificant. This may indirectly indicate that GVHD progression was not B-cell-dependent [25].

In the case of successful engraftment, the atrophy of BM and spleen tissues that develops over time has a direct impact on the severity of GVHD. Moreover, the severity of most blood pathologies depends not only on the type and concentration of the graft but also on the degree of mouse chimerism. Using ANOVA, both the presence and absence of a relationship between the type and concentration of the graft and the dynamics of individual CBC parameters were determined (Table 14).

The continuous decrease in RBC in 2.5 × 10^6^ hT-cells/hPBMC mice throughout the study allowed us to establish a statistically significant relationship between the type and concentration of graft and RBC. At the same time, in 2.5 × 10^6^ hPBMCs mice, an increase in RBC was registered at 68 d.p.i. Probably for this reason, statistical significance was not confirmed. For the same reason, the effect of the type and concentration of the graft on HCT in these mice was not confirmed.

Despite the fact that Mon# and Gran# of 2.5 × 10^6^ hPBMCs mice reached a maximum at 47 d.p.i., statistical significance was confirmed only for Mon#. After reaching peak values, Gran# sharply decreased, approaching the values of control mice, where it remained until the end of the study. At the same time, the dynamics of Mon# had a wave-like decreasing trend. In turn, Mon# of 2.5 × 10^6^ hT-cells/hPBMCs mice reached a maximum at 33 d.p.i. and was on a plateau until 54 d.p.i., after which it sharply decreased. However, Gran# of these mice, having reached a maximum at 33 d.p.i., smoothly decreased until the end of the study.

Verma B. and Wesa A., in a study on hPBMCs engraftment into 4- to 7-week-old female NOG mice, did not perform preliminary myeloablation due to, as stated by the authors, the risk of premature development of severe GVHD. After intravenous administration of 10 × 10^6^ hPBMCs, mice did not develop signs of GVHD for 40 days [12]. This is in sharp contrast to our results, according to which mice that received 10 × 10^6^ hPBMCs were completely eliminated from the study at 36 d.p.i.

Target organ analysis demonstrated a predominance of relative weights of the liver, spleen, lungs, kidneys, and brain in humanized mice over control mice, but not reproductive organs.

We have identified a dependence of the occurrence and development of severe multiorgan pathology on the type and concentration of the graft. The most pronounced BM atrophy was observed in hT-cells/hPBMCs mice compared to hPBMCs mice. This was probably due to the greater antigenicity of the graft.

While 2.5 × 10^6^ hT-cells/hPBMCs mice developed mild splenic white pulp atrophy, 10 × 10^6^ hPBMCs and 10 × 10^6^ hT-cells/hPBMCs mice developed severe parenchymal atrophy accompanied by varying degrees of fibrosis.

The results of our study demonstrated the ability of hCD4^+^-cells not only to circulate in the bloodstream for a long time but also to migrate into the BM, spleen parenchyma, perivascular interstitium of the liver and kidneys, perivascular and peribronchial interstitium of the lungs, lamina propria of the small intestine, endometrium, and various parts of the brain, as well as mesenteric lymph nodes. This prevails over the results presented in Table 13. However, we do not exclude that colleagues simply did not conduct such a large-scale detection of HCs but limited themselves to only some organs. Therefore, the restoration of the human immune system in C-NKG mice by engraftment of hPBMCs or hT-cells mixed with hPBMCs can achieve maximum translatability during experimental HIV infection.

In addition, we found a high positive correlation between:

- the degree of chimerism and GVHD progression in 2.5 × 10^6^ hPBMCs and 2.5 × 10^6^ hT-cells/hPBMCs mice;
- hT-helper cell concentration and GVHD progression in mice 10 × 10^6^ hPBMCs, 5 × 10^6^ hT-cells/hPBMCs, and 10 × 10^6^ hT-cells/hPBMCs;
- GVHD progression and WBC including Lymph# in 10 × 10^6^ hPBMCs mice;
- the degree of chimerism and WBC including Lymph# in 10 × 10^6^ hPBMCs and 5 × 10^6^ hT-cells/hPBMCs mice;
- the degree of chimerism and Mon# in hPBMCs and 5 × 10^6^ hPBMCs mice;
- the degree of chimerism and Gran# in hPBMCs and 5 × 10^6^ hT-cells/hPBMCs mice.

High negative correlations were found between:

- GVHD progression and RBC in all humanized mice;
- GVHD progression and PLT in 10 × 10^6^ hPBMCs and 10 × 10^6^ hT-cells/hPBMCs mice;
- the degree of chimerism and RBC in 5 × 10^6^ hPBMCs and 10 × 10^6^ hT-cells/hPBMCs mice;
- the degree of chimerism and PLT in 10 × 10^6^ hPBMCs and 10 × 10^6^ hT-cells/hPBMCs mice.

Thus, we have identified crucial aspects for the reconstitution of the human immune system in C-NKG mice that affect both the early development of GVHD and the cell engraftment. Using our results, any research group involved in the development of mice with a human immune system will be able to predict the correctness of the conditions chosen for humanization at the early stages of the study.

## 5. Conclusions

Myeloablation and the type and concentration of the graft are critical in the development of humanized immune system C-NKG mouse models. Proper irradiation dose and graft concentration result in prolonged circulation of HCs in mice. This is achieved by delaying the onset of GVHD, extending “the observation window”. Overall, 2.5 × 10^6^ hPBMCs and 2.5 × 10^6^ hT-cells/hPBMCs mice showed engraftment rates of ~55–77% and ~66–78%, respectively, at 82 d.p.i. However, 82 days is not the limit of observation for these mice.

hCD4^+^-cell infiltrates were detected in BM, perivascular interstitium of the liver and kidneys, splenic parenchyma, perivascular and peribronchial interstitium of the lungs, lamina propria of the small intestine, endometrium, brain, and mesenteric lymph nodes.

It would be incorrect to claim that there is a reliable positive correlation between the level of chimerism and the progression of GVHD based on statistical data alone. However, this is supported by published studies demonstrating a direct effect of the major histocompatibility complex (MHC) of donor cells on the progression of GVHD [22]. Equally important is that statistical analysis demonstrated a negative correlation between the degree of chimerism and RBC and PLT. That is, the severity of myelopathy developing within GVHD directly depends on the number of engrafted cells. These results can be scaled up in modeling HIV infection in silico or used as training materials for artificial intelligence.

In Table 13, we provide several examples of humanization of NSG mice with hPBMCs to demonstrate the validity of our humanization protocol. However, different authors adhere to different criteria for assessing the success of humanization or assessing GVHD. In our opinion, primary and secondary factors influencing the progression of GVHD can be distinguished. The primary factors are the genotype of mice and the type of graft, and the secondary factors are the sex and age of mice, the method of myeloablation, and the concentration of the graft. It is worth mentioning that minor histocompatibility antigens (miHAs), the level of cytokines, and the individual microbiome of mice can affect the progression of GVHD. The genotype determines the number of MHC-I molecules, which is the main target for the graft. Michael A. Brehm et al. demonstrated that NSG mice deficient in MHC-I expression do not show GVHD progression over a long period of time after hPBMC transplantation [22].

On the other hand, donor cells also have their own MHC, part of which is human leukocyte antigen (HLA). However, the only natural cells that do not have HLA are erythrocytes. A promising direction seems to be knocking out HLA in pluripotent stem cells using the CRISPR/Cas9 gene editing system [26] and their subsequent engraftment to transgenic mice deficient in MHC-I expression. However, this technology is still imperfect and difficult to scale up. Some studies have shown that human cells engraft better in females than in males [27]. In the future, this may allow us to evaluate the effectiveness of different routes of HIV administration, including vaginal.

For our study, it was important not only to determine the optimal conditions but also to understand the duration and effectiveness of humanization. Since the next stage of our work will be infecting humanized mice with HIV, we were equally interested in the dynamics of the number of hT-helper cells.

Injecting HCs directly into the bloodstream is the fastest delivery method. Intraperitoneal or intracardiac administration can also be used. However, the former may not provide a high humanization effect, and the latter is quite traumatic for animals. In addition, both of these methods require special control of cell administration at the time of injection.

Using the BLT-humanized NSG mice, it was demonstrated that hCD4^+^-cells are capable of independently transporting HIV from the periphery to the brain [28]. However, even with effective antiretroviral therapy, HIV persists in resting hCD4^+^-cells [29]. In turn, we demonstrated the migration of hCD4^+^-cells to lymphoid organs and the brain, which are the main reservoirs in the latent HIV infection. This alone puts hPBMC-humanized mice in a more advantageous position. In the model of latent HIV infection described by Nina C. Flerin et al. NSG mice were humanized by intrasplenic injection of hPBMCs from long-term antiretroviral therapy-suppressed HIV-infected donors [30]. Although the model has demonstrated its viability, its disadvantage is that only one of the latent HIV reservoirs was used. In addition, intrasplenic injection requires special equipment to control the administration. Infecting humanized C-NKG mice with HIV may yield similar results.

A limitation for hPBMC-humanized C-NKG mice may be the earlier progression of GVHD, which is also characteristic of hHSC-chimeric mice. At the same time, the use of hT-cells mixed with autologous hPBMCs is likely to be of limited benefit, since it already contains activated hT-cells. We expect that infection of 2.5 × 10^6^ hPBMCs mice with HIV at 19–26 d.p.i. will also yield good results.

The availability of certain humanized mouse models is closely related to the availability of materials used for humanization. Many countries have strict restrictions or prohibitions on the use of human embryonic stem cells or tissues [31], which do not apply to hPBMCs. In addition, stem cells and embryonic tissues are specific materials that are not always available in the required quantities. Therefore, the use of BLT and HSC models for HIV infection is difficult. We also noted above that the BLT model requires labor-intensive surgical manipulation and a long period of time to restore human cells. The HSC model is much simpler to implement, as mice have multilineage hematopoiesis, so it is possible to study the primary immune response [32].

Our models are unable to reconstitute human multilineage hematopoiesis. This will likely limit the duration of observation after HIV-1 infection to several weeks. However, this is quite sufficient to develop a method for using CRISPR/Cas9 for therapeutic purposes.

Therefore, C-NKG mice, when subjected to optimal preliminary myeloablation and engraftment of hPBMCs or hT-cells mixed with hPBMCs, may be a suitable model for studying HIV infection or for use in preclinical trials.

In addition, in our study, all grafts were cryopreserved and then thawed. Considering that HIV requires, on average, 52 h from the moment of virion export in one generation to export in the next [33], our proposed protocols for engraftment of hPBMCs and hT-cells mixed with hPBMCs may prove successful specifically for gene therapy studies.

## Figures and Tables

**Table 1:** Characteristics of the grafts.

Type of the Graft	Animal Groups
2.5 × 10^6^ hPBMCs	2.5 × 10^6^ hPBMCs
5 × 10^6^ hPBMCs	5 × 10^6^ hPBMCs
10 × 10^6^ hPBMCs	10 × 10^6^ hPBMCs
1.25 × 10^6^ hT-cells mixed with 1.25 × 10^6^ hPBMCs	2.5 × 10^6^ hT-cells/hPBMCs
2.5 × 10^6^ hT-cells mixed with 2.5 × 10^6^ hPBMCs	5 × 10^6^ hT-cells/hPBMCs
5 × 10^6^ hT-cells mixed with 5 × 10^6^ hPBMCs	10 × 10^6^ hT-cells/hPBMCs
1× DPBS	Control

**Table 2:** Fluorescent antibody panel for graft characterization.

Dye/Antibody Conjugate	Catalogue Number
EV450 Anti-Mouse CD45 Antibody [30-F11], 500 T	E-AB-F1136Q
PE/TR Anti-Human CD3 Antibody [OKT-3], 200 T	E-AB-F1001P
PE/Cyanine7 Anti-Human CD4 Antibody [RPA-T4], 200 T	E-AB-F1109H
7-AAD Staining Solution	130-111-568

**Table 3:** Interpretation of *r* and *rs* values.

*r* Value	Interpretation of Correlation
0.75 to 1.00 (−0.75 to −1.00)	Very high positive (negative)
0.50 to 0.75 (−0.50 to −0.75)	High positive (negative)
0.25 to 0.50 (−0.25 to −0.50)	Moderate positive (negative)
0.00 to 0.25 (0.00 to −0.25)	Low positive (negative)

**Table 4:** Analysis of the effect of the type and concentration of the graft on the dynamics of body weight.

Type of the Graft	% of Total Variation	*p*-Value
hPBMCs	21.4	0.0009
hT-cells/hPBMCs	16.79	0.0206

**Table 5:** Analysis of the effect of the type and concentration of the graft on the progression of GVHD.

Type of the Graft	% of Total Variation	*p*-Value
hPBMCs	46.87	<0.0001
hT-cells/hPBMCs	21.54	<0.0001

**Table 6:** Survival analysis.

Mouse Group	Type of the Graft	Log-Rank (Mantel–Cox) Test	Gehan–Breslow–Wilcoxon Test
hPBMCs	Chi square	24.1	22.72
	df	3	3
	*p*-value	<0.0001	<0.0001
hT-cells/hPBMCs	Chi square	15.92	15.28
	df	3	3
	*p*-value	0.0012	0.0016

**Table 7:** Linear correlation between the degree of chimerism and GVHD progression.

Type of the Graft	*r*	*p*-Value
2.5 × 10^6^ hPBMCs	0.72	0.01
5 × 10^6^ hPBMCs	0.53	0.23
10 × 10^6^ hPBMCs	0.49	0.51
2.5 × 10^6^ hT-cells/hPBMCs	0.58	0.06
5 × 10^6^ hT-cells/hPBMCs	0.41	0.36
10 × 10^6^ hT-cells/hPBMCs	0.57	0.18

High positive correlation was found in 2.5 × 10^6^ hPBMCs and 2.5 × 10^6^ hT-cells/hPBMCs mice, but only the result of 2.5 × 10^6^ hPBMCs mice was statistically significant.

**Table 8:** Linear correlation between hT-helper cells concentration and GVHD progression.

Type of the Graft	*r*	*p*-Value
2.5 × 10^6^ hPBMCs	−0.59	0.06
5 × 10^6^ hPBMCs	0.24	0.60
10 × 10^6^ hPBMCs	0.95	0.05
2.5 × 10^6^ hT-cells/hPBMCs	0.03	0.94
5 × 10^6^ hT-cells/hPBMCs	0.77	0.04
10 × 10^6^ hT-cells/hPBMCs	0.73	0.06

High negative correlation was observed only in 2.5 × 10^6^ hPBMCs mice, while a high positive correlation was confirmed in 10 × 10^6^ hPBMCs and 5 × 10^6^ hT-cells/hPBMCs mice. However, only the result of 5 × 10^6^ hT-cells/hPBMCs mice was statistically significant.

**Table 9:** Linear correlation between blood count and GVHD progression.

Type of the Graft	WBC	RBC	PLT	Lymph#	Mon#	Gran#
2.5 × 10^6^ hPBMCs	*r* = 0.11; *p* = 0.74	*r* = −0.80; *p* = 0.003	*r* = −0.54; *p* = 0.08	*r* = −0.01; *p* = 0.98	*r* = 0.42; *p* = 0.19	*r* = 0.17; *p* = 0.62
5 × 10^6^ hPBMCs	*r* = −0.21; *p* = 0.66	*r* = −0.83; *p* = 0.02	*r* = −0.33; *p* = 0.47	*r* = −0.24; *p* = 0.61	*r* = 0.04; *p* = 0.93	*r* = 0.19; *p* = 0.68
10 × 10^6^ hPBMCs	*r* = 0.58; *p* = 0.42	*r* = −0.79; *p* = 0.21	*r* = −0.66; *p* = 0.34	*r* = 0.79; *p* = 0.21	*r* = 0.12; *p* = 0.88	*r* = 0.44; *p* = 0.56
2.5 × 10^6^ hT-cells/hPBMCs	*r* = −0.19; *p* = 0.57	*r* = −0.95; *p* = 0.00001	*r* = −0.73; *p* = 0.01	*r* = 0.01; *p* = 0.99	*r* = −0.14; *p* = 0.67	*r* = −0.29; *p* = 0.39
5 × 10^6^ hT-cells/hPBMCs	*r* = 0.12; *p* = 0.80	*r* = −0.91; *p* = 0.005	*r* = −0.12; *p* = 0.80	*r* = 0.13; *p* = 0.78	*r* = 0.18; *p* = 0.70	*r* = 0.12; *p* = 0.80
10 × 10^6^ hT-cells/hPBMCs	*r* = 0.07; *p* = 0.89	*r* = −0.83; *p* = 0.02	*r* = −0.72; *p* = 0.07	*r* = 0.26; *p* = 0.58	*r* = −0.17; *p* = 0.71	*r* = −0.15; *p* = 0.75

High positive correlations between GVHD progression and WBC were confirmed in 10 × 10^6^ hPBMCs mice, but not in any hT-cells/hPBMCs mice; very high negative correlations between GVHD progression and RBC were found in all mice, but statistically significant results were found in 2.5 × 10^6^ hPBMCs, 5 × 10^6^ hPBMCs, 2.5 × 10^6^ hT-cells/hPBMCs, and 10 × 10^6^ hT-cells/hPBMCs mice; negative correlations between GVHD progression and PLT were confirmed in all mice, but statistically significant results were found only in 2.5 × 10^6^ hT-cells/hPBMCs mice; a high positive correlation between GVHD progression and Lymph# of 10 × 10^6^ hPBMCs mice was found; however, statistical significance was not determined for either group; a moderate positive correlation between GVHD progression and Mon# of 2.5 × 10^6^ hPBMCs mice was found; however, statistical significance was not determined for either group; a high positive correlation between GVHD progression and Gran# of 10 × 10^6^ hPBMCs mice was confirmed; however, statistical significance was not determined for either group.

**Table 10:** Linear correlation between the degree of chimerism and the count of blood cell populations.

Type of the Graft	WBC	RBC	PLT	Lymph#	Mon#	Gran#
2.5 × 10^6^ hPBMCs	*r* = 0.45; *p* = 0.17	*r* = −0.68; *p* = 0.02	*r* = −0.46; *p* = 0.15	*r* = 0.06; *p* = 0.85	*r* = 0.69; *p* = 0.02	*r* = 0.67; *p* = 0.03
5 × 10^6^ hPBMCs	*r* = 0.57; *p* = 0.18	*r* = −0.70; *p* = 0.08	*r* = −0.11; *p* = 0.81	*r* = 0.30; *p* = 0.52	*r* = 0.73; *p* = 0.06	*r* = 0.82; *p* = 0.02
10 × 10^6^ hPBMCs	*r* = 0.97; *p* = 0.03	*r* = −0.55; *p* = 0.45	*r* = −0.69; *p* = 0.31	*r* = 0.84; *p* = 0.16	*r* = 0.89; *p* = 0.11	*r* = 1.00; *p* = 0.004
2.5 × 10^6^ hT-cells/hPBMCs	*r* = 0.52; *p* = 0.10	*r* = −0.64; *p* = 0.03	*r* = −0.08; *p* = 0.81	*r* = 0.52; *p* = 0.10	*r* = 0.38; *p* = 0.25	*r* = 0.36; *p* = 0.27
5 × 10^6^ hT-cells/hPBMCs	*r* = 0.80; *p* = 0.03	*r* = −0.26; *p* = 0.57	*r* = 0.05; *p* = 0.91	*r* = 0.84; *p* = 0.02	*r* = 0.65; *p* = 0.11	*r* = 0.77; *p* = 0.04
10 × 10^6^ hT-cells/hPBMCs	*r* = 0.53; *p* = 0.22	*r* = −0.74; *p* = 0.06	*r* = −0.89; *p* = 0.01	*r* = 0.46; *p* = 0.30	*r* = 0.43; *p* = 0.33	*r* = 0.49; *p* = 0.26

Statistically significant very high positive correlation between the degree of chimerism and WBC of 10 × 10^6^ hPBMCs and 5 × 10^6^ hT-cells/hPBMCs mice was determined; negative correlation between the degree of chimerism and RBC for all mice was determined; however, statistically significant results were 2.5 × 10^6^ hPBMCs and 2.5 × 10^6^ hT-cells/hPBMCs; high and very high negative correlations between the degree of chimerism and PLT of 10 × 10^6^ hPBMCs and 10 × 10^6^ hT-cells/hPBMCs mice, respectively, were confirmed; however, statistical significance was only in 5 × 10^6^ hT-cells/hPBMCs mice; a very high positive correlations between the degree of chimerism and Lymph# of 10 × 10^6^ hPBMCs and 5 × 10^6^ hT-cells/hPBMCs mice were determined; however, only the results of 5 × 10^6^ hT-cells/hPBMCs mice had statistical significance; a very high and high positive correlations between the degree of chimerism and Mon# of hPBMCs mice, as well as a very high one in 5 × 10^6^ hT-cells/hPBMCs mice, were determined; however, only the results of 2.5 × 10^6^ hPBMCs mice had statistical significance; statistically significant very high and high positive correlations between the degree of chimerism and Gran# of hPBMCs mice and a high one in 5 × 10^6^ hT-cells/hPBMCs mice were confirmed.

**Table 11:** The most common macromorphological pathologies.

Pathology	Mice Count
Anemia of the spleen	17
Anemia of the liver	15
Myocardial anemia	14
Anemia of the kidneys	12
Uterine and ovarian atrophy	10
Atrophy of the spleen	9
Liver atrophy	7
Hyperplasia of the spleen	5

**Table 12:** Correlation between the number of hCD4^+^-cells in tissue sections and the type of the graft.

Type of the Graft	BM	Spleen	Kidneys	Liver	Lungs	Small Intestine	Uterus	Brain
hPBMCs	*rs* = 0.5 *p* = 1.0	*rs* = 0.5 *p* = 1.0	*rs* = −1.0 *p* = 0.3	*rs* = 0 *p* = 1.0	*rs* = 0.5 *p* = 1.0	*rs* = 0.5 *p* = 1.0	*rs* = −0.5 *p* = 1.0	*rs* = −1.0 *p* = 0.3
hT-cells/PBMCs	*rs* = 0.5 *p* = 1.0	*rs* = −1.0 *p* = 0.3	*rs* = −0.5 *p* = 1.0	*rs* = 0.9 *p* = 0.7	*rs* = 0.5 *p* = 1.0	*rs* = 0 *p* = 1.0	*rs* = −1.0 *p* = 0.3	*rs* = 0 *p* = 1.0

A very high positive correlation was determined only for hCD4^+^-cells in the liver of hT-cells/hPBMCs mice. A moderate positive correlation was confirmed for hCD4^+^-cells in the bone marrow and lungs of all mice, as well as in the spleen and small intestine of hPBMCs mice. A very high negative correlation was determined for hCD4^+^-cells in the kidneys and brain of hPBMCs mice, as well as in the spleen and uterus of hT-cells/hPBMCs mice. A moderate negative correlation was confirmed for hCD4^+^-cells in the uterus of hPBMCs mice and in the kidneys of hT-cells/hPBMCs mice.

**Table 13:** Comparative characteristics of hPBMCs-engrafted NSG mice.

Concentration of hPBMCs and Route of Administration	hCD3^+^ T-Cells Degree of Engraftment, %	Duration of Observation, d.p.i.	Graft Localization	References
10 × 10^6^, intraperitoneally	~62–79	112	Peripheral blood, lymph nodes, spleen, liver	[20]
10 × 10^6^, intravenously	No data	42	Peripheral blood, skin, lungs, liver, eyes, nose, ear, tongue, salivary glands, brain	[21]
10 × 10^6^, intravenously	~80–99	~84–119	Peripheral blood, spleen	[22]
10 × 10^6^, intravenously	~85	35	Peripheral blood	[23]
10 × 10^6^, intravenously	~95–98	49	Peripheral blood, spleen, lymph node, bone marrow	[24]

**Table 14:** The relationship between the CBC index and the type of graft.

CBC Index	The Type of the Graft	
	2.5 × 10^6^ hPBMCs	2.5 × 10^6^ hT-Cells/hPBMCs
WBC	No	No
RBC	No	Yes
HGB	Yes	Yes
HCT	No	Yes
MCV	Yes	Yes
MCH	No	No
MCHC	Yes	Yes
RDW	Yes	Yes
PLT	Yes	Yes
MPV	Yes	Yes
PDW	No	No
PCT	Yes	Yes
Lymph %	No	No
Mon %	Yes	Yes
Gran %	No	No
Lymph #	No	No
Mon #	Yes	No
Gran #	No	Yes

## Data Availability

The authors confirm that the data supporting the findings of this study are available within the manuscript.

## Supplementary Materials

The following are available online at https://www.mdpi.com/article/10.3390/life15071129/s1, Figure S1: Strategy for defining hT-cell and hT-helper cell populations relative to mouse lymphocytes. Figure S2: Effect of the type and concentration of the graft on the dynamics of leukocyte indices. Figure S3: Effect of the type and concentration of the graft on the dynamics of red blood cell indices. Figure S4: Effect of the type and concentration of the graft on platelet dynamics. Table S1: The fraction of hT-helper cells in the T-cell population over time.
