# Supplementary material for: Human Immune System Reconstitution in NOD/Shi-Prkdcscid Il2rgem1/Cyagen Mice to Study HIV Infection: Challenges and Pitfalls
Source: Life (Basel). 2025 Jul 18;15(7):1129. doi: 10.3390/life15071129 (PMC12300024; doi:10.3390/life15071129)
Supplement: Supplementary file 1 [file life-15-01129-s001.zip › Figure S1. Strategy for defining hT-cell and hT-helper cell populations relative to mouse lymphocytes.pdf]

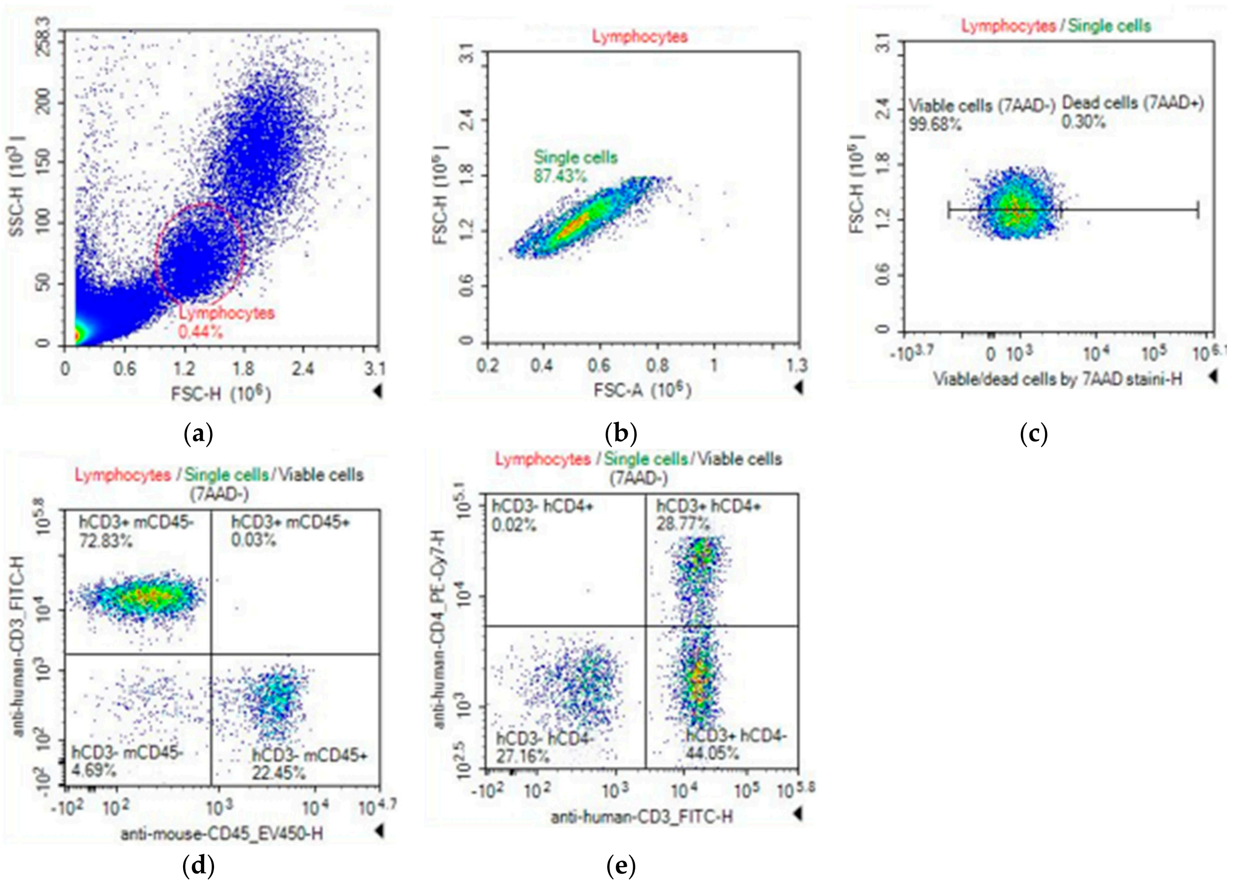


**Figure S1.** Strategy for defining hT-cell and hT-helper cell populations relative to mouse lymphocytes. (a) Determination of the lymphocyte fraction by FSC and SSC; (b) Single-cell cytometry; (c) Selection of 7 AAD undyed living cells; (d) Separation of populations of hT-cells ($hCD3^{+}mCD45^{-}$) and mouse lymphocytes ($hCD3^{-}mCD45^{+}$); (e) Determination of the fraction of hT-helper cells ($hCD3^{+}hCD4^{+}$) in the hT-cells population.
