# Supplementary material for: Human Immune System Reconstitution in NOD/Shi-Prkdcscid Il2rgem1/Cyagen Mice to Study HIV Infection: Challenges and Pitfalls
Source: Life (Basel). 2025 Jul 18;15(7):1129. doi: 10.3390/life15071129 (PMC12300024; doi:10.3390/life15071129)
Supplement: Supplementary file 1 [file life-15-01129-s001.zip › Figure S2. Effect of the type and concentration of the graft on the dynamics of leukocyte indices. Revised.pdf]

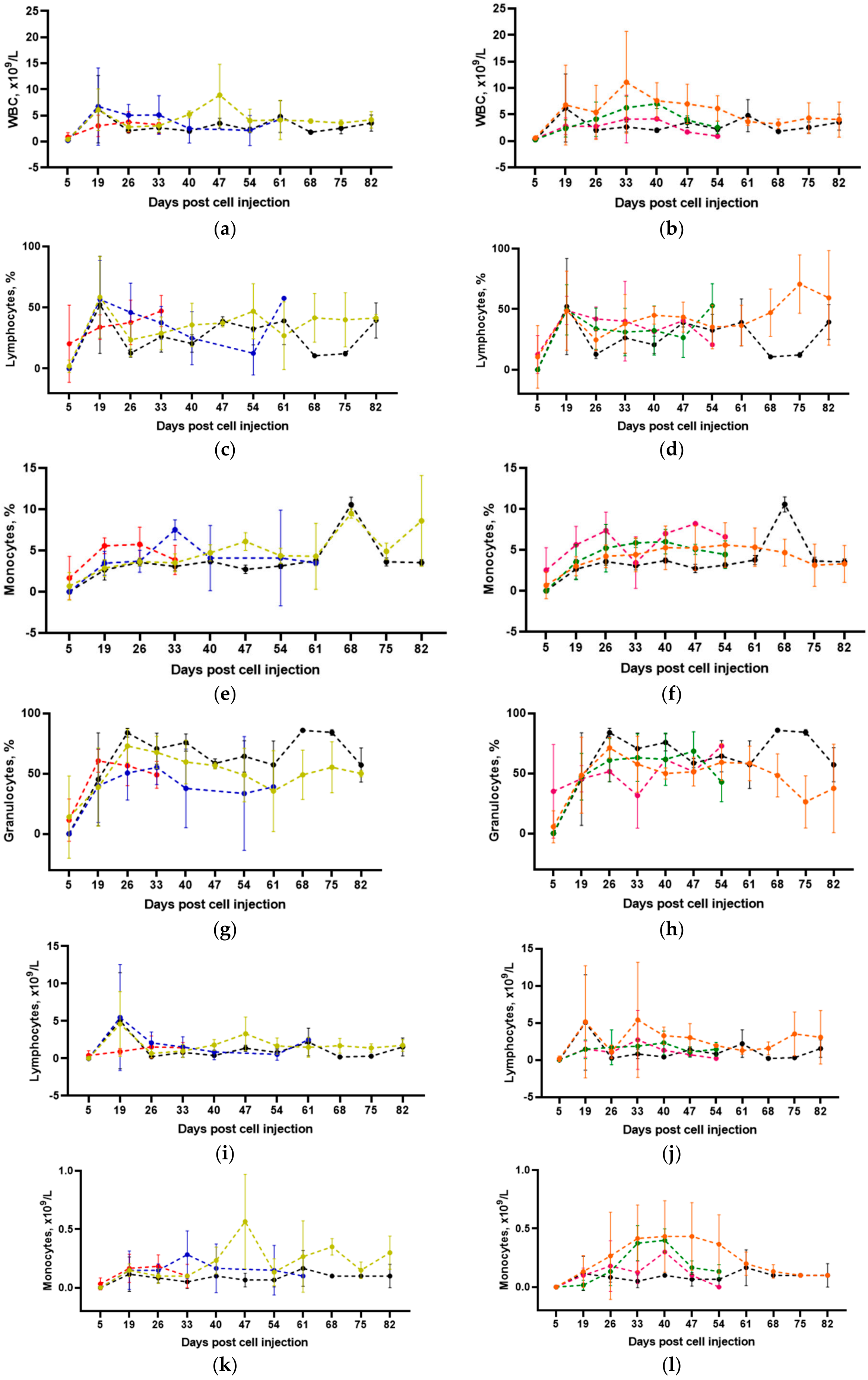

WBC, x10^9/L
Days post cell injection
(a)
WBC, x10^9/L
Days post cell injection
(b)
Lymphocytes, %
Days post cell injection
(c)
Lymphocytes, %
Days post cell injection
(d)
Monocytes, %
Days post cell injection
(e)
Monocytes, %
Days post cell injection
(f)
Granulocytes, %
Days post cell injection
(g)
Granulocytes, %
Days post cell injection
(h)
Lymphocytes, x10^9/L
Days post cell injection
(i)
Lymphocytes, x10^9/L
Days post cell injection
(j)
Monocytes, x10^9/L
Days post cell injection
(k)
Monocytes, x10^9/L
Days post cell injection
(l)

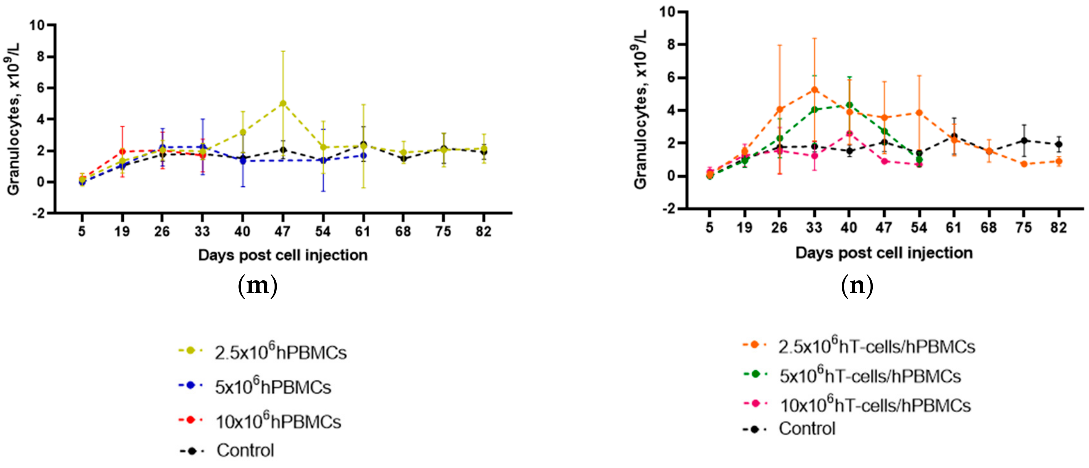

Figure S2. Effect of the type and concentration of the graft on the dynamics of leukocyte indices. (a) WBC of 2.5×10^6 hPBMCs mice reached a plateau at 54 d.p.i.; (b) WBC of 2.5×10^6 hT-cells/hPBMCs mice reached a plateau at 61 d.p.i.; (c) Lymph% dynamics in 2.5×10^6 hPBMCs mice was undulating throughout the study; (d) In 2.5×10^6 hT-cells/hPBMCs mice, pronounced relative lymphocytosis was observed from 61 to 82 d.p.i.; (e) Low relative monocytosis was observed in all hPBMCs mice; (f) By the end of the study, Mon% of 2.5×10^6 hT-cells/hPBMCs mice was almost equal to that of the control mice; (g) Relative granulocytopenia observed in 2.5×10^6 hPBMCs mice reached control mice values by the end of the study; (h) Relative granulocytopenia was observed in all hT-cells/hPBMCs mice throughout the study; (i) More pronounced absolute lymphocytosis was in 2.5×10^6 hPBMCs mice; (j) More pronounced absolute lymphocytosis was in 2.5×10^6 hT-cells/hPBMCs mice; (k) More pronounced absolute monocytosis was in 2.5×10^6 hPBMCs mice; (l) The most pronounced absolute monocytosis was in 2.5×10^6 hT-cells/hPBMCs mice. However, by the end of the study, Mon# was equal to that of the control mice; (m) The dynamics of Gran# in hPBMCs mice were practically no different from that in the control mice; (n) In 2.5×10^6 hT-cells/hPBMCs and 5×10^6 hT-cells/hPBMCs mice, pronounced absolute granulocytosis was observed from 19 to 54 d.p.i.
