# Supplementary material for: Human Immune System Reconstitution in NOD/Shi-Prkdcscid Il2rgem1/Cyagen Mice to Study HIV Infection: Challenges and Pitfalls
Source: Life (Basel). 2025 Jul 18;15(7):1129. doi: 10.3390/life15071129 (PMC12300024; doi:10.3390/life15071129)
Supplement: Supplementary file 1 [file life-15-01129-s001.zip › Figure S3. Effect of the type and concentration of the graft on the dynamics of red blood cell indices. Revised.pdf]

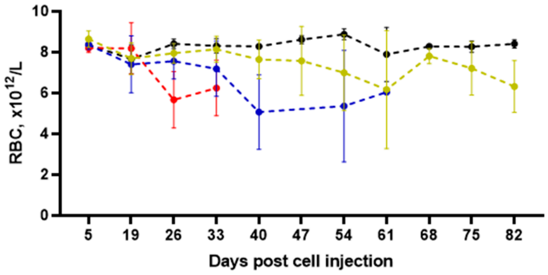

(a)

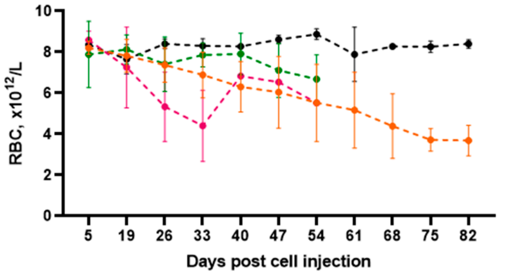

(b)

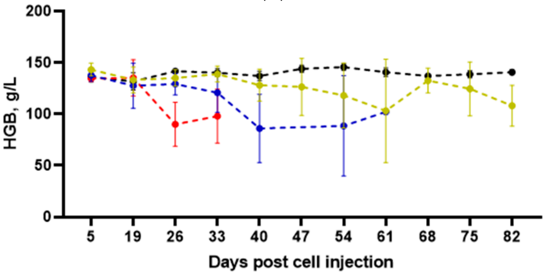

(c)

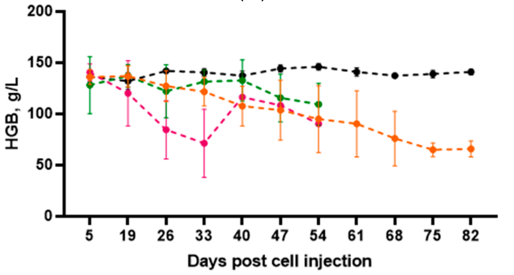

(d)

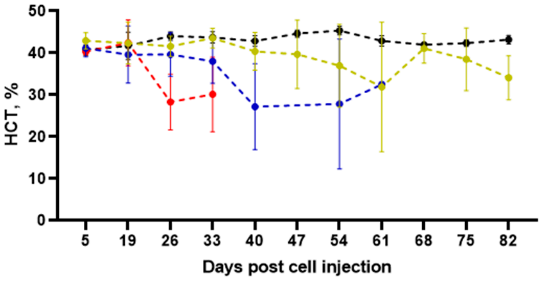

(e)

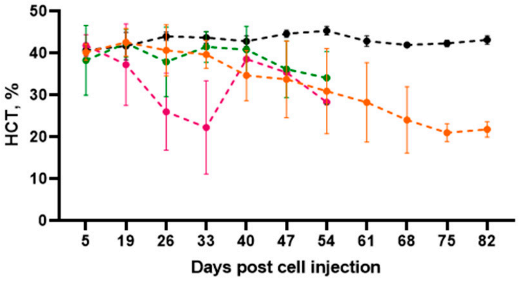

(f)

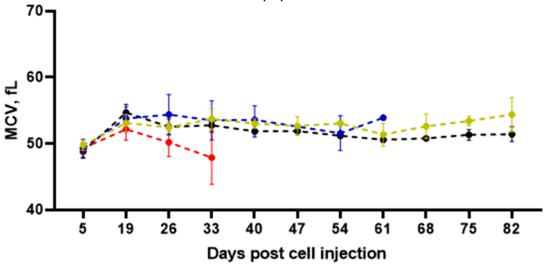

(g)

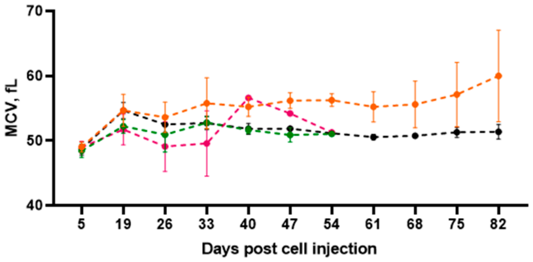

(h)

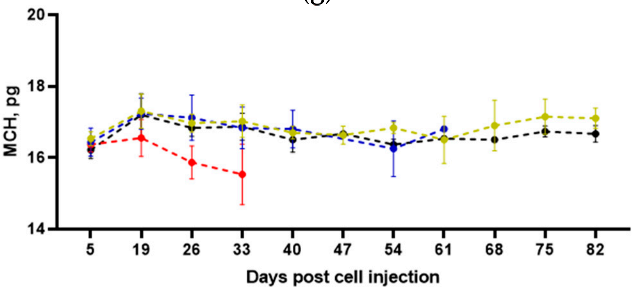

(i)

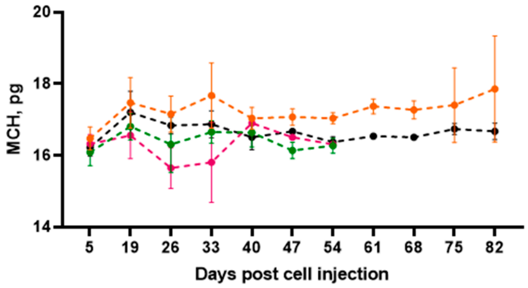

(j)

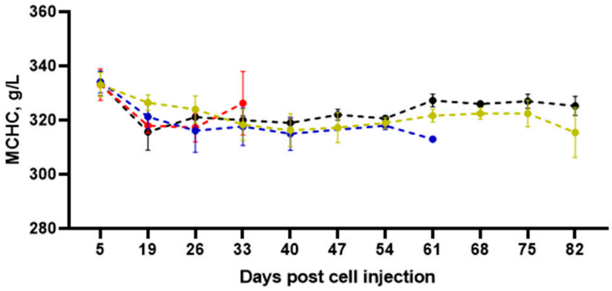

(k)

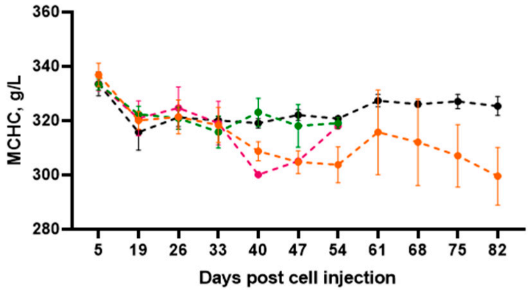

(l)

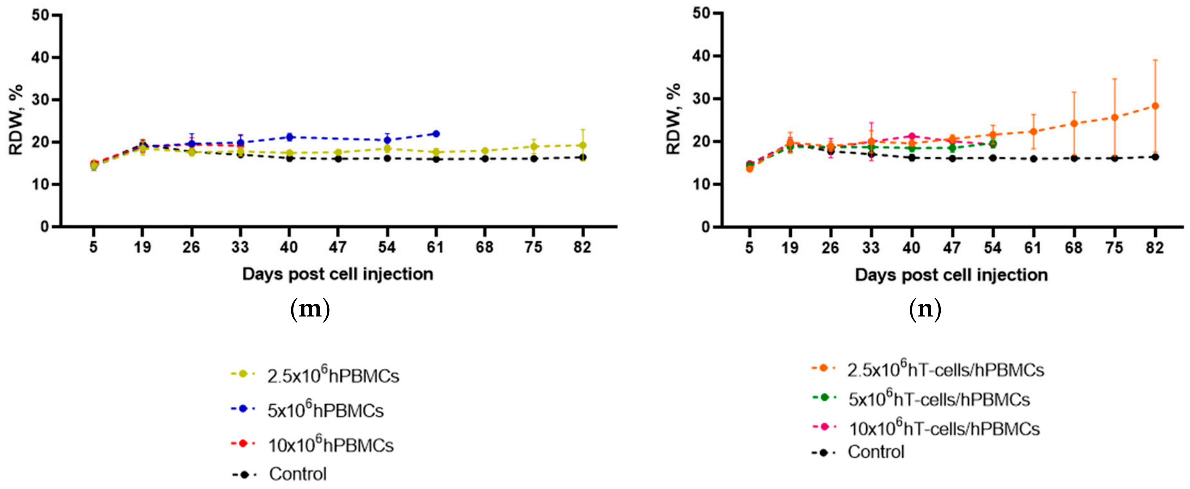


**Figure S3.** Effect of the type and concentration of the graft on the dynamics of red blood cell indices. (a, c, e) Anemia was recorded in all hPBMCs mice at 19 d.p.i. However, a significant correction was observed in 2.5×10$^6$ hPBMCs mice at 68 d.p.i.; (b, d, f) Progressive anemia was recorded in all hT-cells/hPBMCs mice at 19 d.p.i.; (g-j) MCV and MCH values of 2.5×10$^6$ hPBMCs and 2.5×10$^6$ hT-cells/hPBMCs mice slightly exceeded those of other groups throughout the study; (k, l) MCHC values of humanized mice were generally lower than those of controls; (m) 2.5×10$^6$ hPBMCs mice had the lowest RDW values throughout the study; (n) 2.5×10$^6$ hT-cells/hPBMCs mice showed increasing RDW values over time until the end of the study.
