# Supplementary material for: Human Immune System Reconstitution in NOD/Shi-Prkdcscid Il2rgem1/Cyagen Mice to Study HIV Infection: Challenges and Pitfalls
Source: Life (Basel). 2025 Jul 18;15(7):1129. doi: 10.3390/life15071129 (PMC12300024; doi:10.3390/life15071129)
Supplement: Supplementary file 1 [file life-15-01129-s001.zip › Figure S4. Effect of the type and concentration of the graft on platelet dynamics. Revised.pdf]

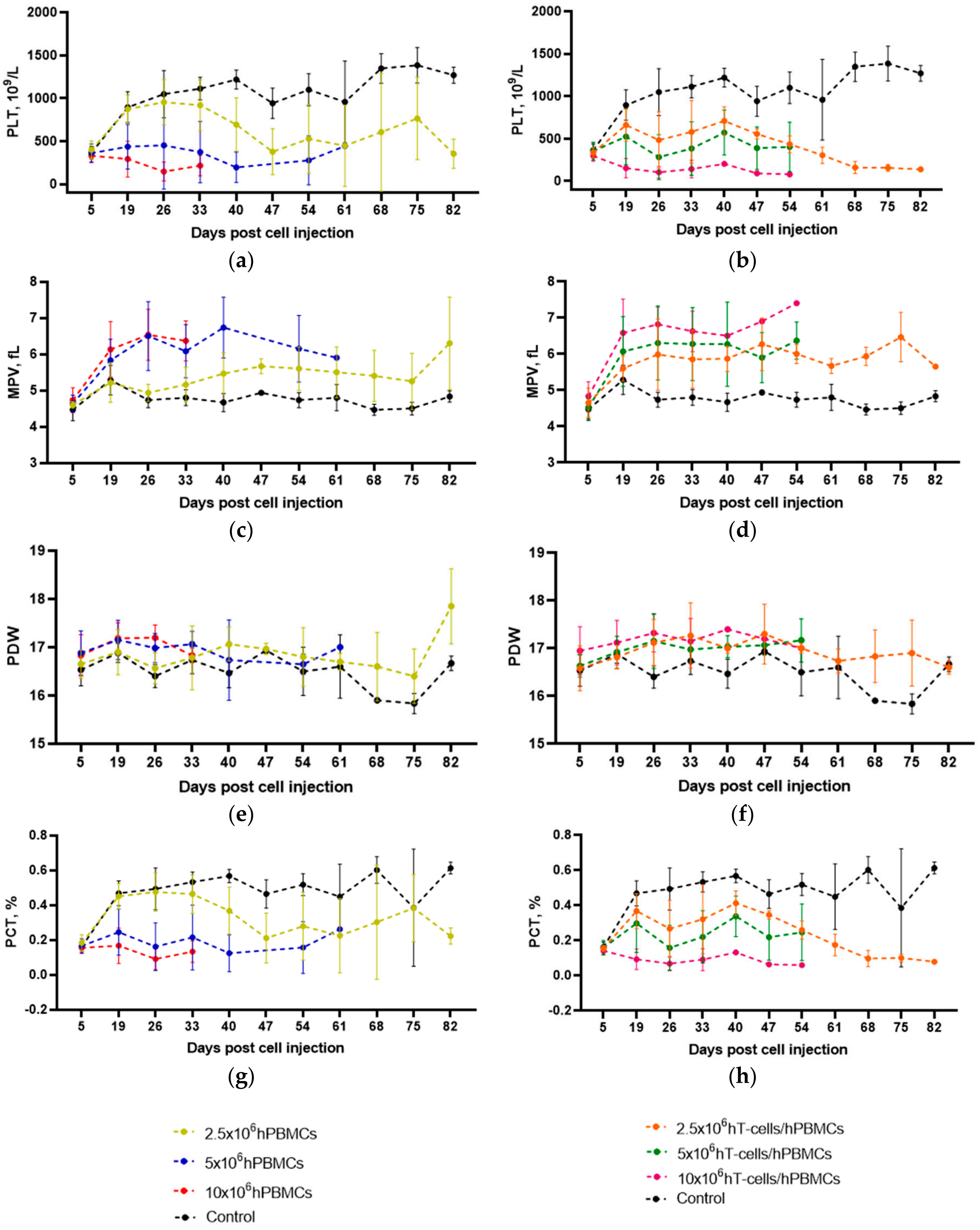

Figure S4. Effect of the type and concentration of the graft on platelet dynamics. (a, b) PLT dynamics and the initial concentration of grafts were inversely correlated; (c, d) MPV dynamics and the initial number of administered HCs were directly correlated; (e, f) PDW dynamics did not depend on the initial concentration of HCs; (g, h) High initial concentration of graft significantly reduce recipient's PCT.
