# Supplementary material for: Human Immune System Reconstitution in NOD/Shi-Prkdcscid Il2rgem1/Cyagen Mice to Study HIV Infection: Challenges and Pitfalls
Source: Life (Basel). 2025 Jul 18;15(7):1129. doi: 10.3390/life15071129 (PMC12300024; doi:10.3390/life15071129)
Supplement: Supplementary file 1 [file life-15-01129-s001.zip › Table S1. The fraction of hT-helper cells in the T-cell population over time.pdf]

Table S1. The fraction of hT-helper cells in the T-cell population over time.

D.p.i.	Mean + SD, %					
	**2.5×10^6 hPBMCs**	**5×10^6 hPBMCs**	**10×10^6 hPBMCs**	**2.5×10^6 hT-cells/hPBMCs**	**5×10^6 hT-cells/hPBMCs**	**10×10^7 hT-cells/hPBMCs**
5	34.41392± 32.52008	31.70442± 11.81567	39.471± 18.58146	22.2577± 11.9845	27.49486± 24.88755	34.45127± 6.365055
19	45.41831± 23.60543	35.98485± 2.852882	34.871± 10.91146	43.74669± 12.50433	39.85816± 11.45515	32.00421± 3.138654
26	63.93957± 22.06206	38.65542± 12.60684	43.79826± 14.21148	45.84781± 10.822	44.21831± 19.44339	47.04744± 8.545513
33	66.27167± 19.82305	43.58359± 8.888177	51.10189± 12.00142	55.6011± 11.42767	60.93993± 17.84959	59.84782± 15.90799
40	52.32187± 21.60819	62.21688± 16.44614	N/A	54.51368± 1.62784	54.61076± 2.626395	54.41972±0
47	41.94334± 36.95764	N/A	N/A	54.31702± 3.523159	59.08783± 12.03355	73.94636±0
54	48.18353± 30.35845	41.40831± 0.041012	N/A	40.92644± 19.87899	61.49779± 24.44934	52.48566±0
61	44.94087± 25.83996	35.63057± 0	N/A	44.32368± 16.57691	N/A	N/A
68	11.46851± 8.66205	N/A	N/A	43.36495± 18.25509	N/A	N/A
75	35.5442± 10.94862	N/A	N/A	45.87436± 13.7143	N/A	N/A
82	28.79793± 27.72074	N/A	N/A	36.69889± 22.44528	N/A	N/A
