# Supplementary material for: Human Immune System Reconstitution in NOD/Shi-Prkdcscid Il2rgem1/Cyagen Mice to Study HIV Infection: Challenges and Pitfalls
Source: Life (Basel). 2025 Jul 18;15(7):1129. doi: 10.3390/life15071129 (PMC12300024; doi:10.3390/life15071129)
Supplement: Supplementary file 1 [file life-15-01129-s001.zip › The ARRIVE Guidelines Checklist. Revised.pdf]

Item	Recommendation	Section/line number, or reason for not reporting
Study design	1 For each experiment, provide brief details of study design including:<br>a. The groups being compared, including control groups. If no control group has been used, the rationale should be stated. b. The experimental unit (e.g. a single animal, litter, or cage of animals)	1 a. Each group of animals was injected with grafts of a certain type and concentration. Therefore, the animal groups are shown in Table 1.<br>b. Each group was kept entirely in an individual cage. The groups were isolated from each other. Therefore, the experimental unit was considered to be a cage (group).
Sample size	2 a. Specify the exact number of experimental units allocated to each group, and the total number in each experiment. Also indicate the total number of animals used. b. Explain how the sample size was decided. Provide details of any a priori sample size calculation, if done.	2 a. A total of 7 groups of 6 animals in each group were used. Of these, 6 groups were experimental and one was a control group. Therefore, a total of 42 animals were used in the study.<br>b. The survival rate was used as a basis for determining the sample size. According to the calculations by Sample Size Calculator tool [Arifin, W. N; Zahiruddin, W. M. *Sample size calculation in animal studies using resource equation approach. Malays J Med Sci,* **2017**, *24*, pp. 101-105, https://doi.org/10.21315/mjms2017.24.5.11], the minimum group size was 3 mice. However, we assumed that 50% of the mice in each group would die or be euthanized. Therefore, the size of each group was increased to 6 mice. In addition, a maximum of 6 mice can be kept in one cage. As is known, the illumination level is not the same at different heights. Given the design of the ISOcage P bioexclusion system, a maximum of 6 cages can be placed at one height. Therefore, by placing all the experimental animals in one row, the most identical conditions were created.
Inclusion and exclusion criteria	3 a. Describe any criteria used for including and excluding animals (or experimental units) during the experiment, and data points during the analysis. Specify if these criteria were established a priori. If no criteria	3 a. The criteria for excluding animals from the experiment were assigning a mouse 7 or more GVHD scores and body weight loss >25%. These criteria were established in advance.<br>b. In the analysis of the effect of the type and concentration of the graft on the activation of the immune system of mice, the results of one 2.5×10^6 hPBMCs, one 5×10^6 hPBMCs, three 10×10^6 hPBMCs, one 2.5×10^6 hT-cells/hPBMCs, two 5×10^6 hT-cells/hPBMCs, and five 10×10^6 hPBMCs mice were not included due to their sudden death.<br>c.

<table>
<tr>
<td></td>
<td>were set, state this explicitly.
b. For each experimental group, report any animals, experimental units or data points not included in the analysis and explain why. If there were no exclusions, state so.
c. For each analysis, report the exact value of n in each experimental group.</td>
<td>
<table>
<tr><th>2.5×10^6 hPBMCs, n</th><th>5×10^6 hPBMCs, n</th><th>10×10^6 hPBMCs, n</th><th>2.5×10^6 hT-cells/hPBMCs, n</th><th>5×10^6 hT-cells/hPBMCs, n</th><th>10×10^6 hPBMCs, n</th></tr>
<tr><td colspan="6">Analysis of the effect of graft type and concentration on body weight dynamics</td></tr>
<tr><td>6</td><td>6</td><td>6</td><td>6</td><td>6</td><td>6</td></tr>
<tr><td colspan="6">Analysis of the effect of the type and concentration of the graft on the dynamics of GVHD</td></tr>
<tr><td>6</td><td>6</td><td>6</td><td>6</td><td>6</td><td>6</td></tr>
<tr><td colspan="6">Analysis of the effect of the type and concentration of the graft on survival probability</td></tr>
<tr><td>6</td><td>6</td><td>6</td><td>6</td><td>6</td><td>6</td></tr>
<tr><td colspan="6">Analysis of the individual effects of the type and concentration of the graft on immune activation in mice</td></tr>
<tr><td>5</td><td>5</td><td>3</td><td>5</td><td>4</td><td>1</td></tr>
<tr><td colspan="6">Analysis of the effect of type and concentration of the graft on degree of chimerism</td></tr>
<tr><td>6</td><td>6</td><td>6</td><td>6</td><td>6</td><td>6</td></tr>
<tr><td colspan="6">Analysis of the dynamics of the fraction of hT-helper cells in the T-cell population</td></tr>
<tr><td>6</td><td>6</td><td>6</td><td>6</td><td>6</td><td>6</td></tr>
<tr><td colspan="6">Analysis of the linear correlation between degree of chimerism and GVHD progression</td></tr>
<tr><td>6</td><td>6</td><td>6</td><td>6</td><td>6</td><td>6</td></tr>
<tr><td colspan="6">Analysis of the linear correlation between hT-helper cells concentration and GVHD progression</td></tr>
<tr><td>6</td><td>6</td><td>6</td><td>6</td><td>6</td><td>6</td></tr>
<tr><td colspan="6">Analysis of the linear correlation between blood count and GVHD progression</td></tr>
<tr><td>6</td><td>6</td><td>6</td><td>6</td><td>6</td><td>6</td></tr>
<tr><td colspan="6">Analysis of the linear correlation between the degree of chimerism count of blood cell populations</td></tr>
<tr><td>6</td><td>6</td><td>6</td><td>6</td><td>6</td><td>6</td></tr>
<tr><td colspan="6">Macromorphological analysis</td></tr>
<tr><td>6</td><td>6</td><td>6</td><td>6</td><td>6</td><td>6</td></tr>
<tr><td colspan="6">Analysis of the target organ weights relative to mouse body weight</td></tr>
<tr><td>6</td><td>6</td><td>6</td><td>6</td><td>6</td><td>6</td></tr>
<tr><td colspan="6">Micromorphological examinations</td></tr>
<tr><td>6</td><td>6</td><td>6</td><td>6</td><td>6</td><td>6</td></tr>
</table>
</td>
</tr>
<tr>
<td>Randomisation</td>
<td>4 a. State whether randomization was used to allocate experimental units to control and treatment groups. If done, provide the method used to generate the randomization sequence.
b. Describe the strategy used to minimize potential confounders such as the order of treatments and measurements, or animal/cage location. If confounders were not controlled, state this explicitly.</td>
<td>4 a. Animals were randomized into 7 groups of 6 mice per group using RandoMice v1.1.7 software based on body weight.
b. All animal manipulations were performed under the same conditions. The cages in which experimental groups were kept were located in the same row. GVHD detection was performed by a person who was not aware of the research protocol. All measurements were performed on certified equipment using control samples (where applicable).</td>
</tr>
</table>

Blinding	5 Describe who was aware of the group allocation at the different stages of the experiment (during the allocation, the conduct of the experiment, the outcome assessment, and the data analysis).	5 Only the animal caregivers were aware of the group allocation during the location and the conduct of the experiment.<br>Biological samples obtained from animals and their test results were encoded for specialists who were aware of group allocation.<br>Data analysis was carried out after decoding the results of the study of biological material samples.
Outcome measures	6 a. Clearly define all outcome measures assessed (e.g. cell death, molecular markers, or behavioral changes). b. For hypothesis-testing studies, specify the primary outcome measure, i.e. the outcome measure that was used to determine the sample size.	6 a. The assessed outcome measures were:<br>- median survival >70%;<br>- the number of human cells in the whole blood of experimental mice > 0.<br>b. Not applicable.
Statistical methods	7 a. Provide details of the statistical methods used for each analysis, including software used. b. Describe any methods used to assess whether the data met the assumptions of the statistical approach, and what was done if the assumptions were not met.	7 a. Statistical data processing was performed using the GraphPad Prism 9.0.0 software product. Survival analysis was performed using the Kaplan-Meier method in the modifications of Mantel-Cox and Gehan-Breslow-Wilcoxon. The dynamics of CBC indexes and the interaction of grafts and the recipient *in vivo* were examined by ANOVA, determination of the Pearson linear correlation coefficient (*r*) and Tukey's multiple comparisons test. To determine *r*, we used the median values for the group of mice for the entire observation period. The interpretation of the *r* values is presented in Table 3. The threshold value of the significance level was set at 0.05. If the *p*-value was below the threshold value of the significance level, the result was considered statistically significant.<br>b. If the assumptions were not met, the data was not taken into account.
Experimental animals	8 a. Provide species-appropriate details of the animals used, including species, strain and substrain, sex, age	8 a. 5–6-week-old female NOD/Shi-*Prkdc*^{scid}*Il2rg*^{em1}/Cyagen (C-NKG) mice.<br>b. The provenance of animals: Cyagen Biosciences Inc, Suzhou, China; health/immune status: specific pathogen-free/ immunodeficiency.

	or developmental stage, and, if relevant, weight. b. Provide further relevant information on the provenance of animals, health/immune status, genetic modification status, genotype, and any previous procedures.	
Experimental procedures	9 For each experimental group, including controls, describe the procedures in enough detail to allow others to replicate them, including:<br>a. What was done, how it was done and what was used.<br>b. When and how often.<br>c. Where (including detail of any acclimatization periods).<br>d. Why (provide rationale for procedures)	9 a, b See the "Materials and Methods" section of the manuscript.<br>c. Animal quarantine was carried out in a special isolated room. Acclimatization and maintenance of animals during the experiment were carried out in another room. The animals were kept, and all manipulations were carried out on the same floor in rooms with the same environmental conditions.<br>d. All procedures and manipulations were carried out in analogy with previously published studies that are freely available.
Results	10 For each experiment conducted, including independent replications, report:<br>a. Summary/descriptive statistics for each experimental group, with a measure of variability where applicable (e.g.	10 a. See the Tables 3, 4, 5, 6, 7, 8, 9, 10, 13, and Supplementary table S1.<br>b. Not applicable.

	mean and SD, or median and range). b. If applicable, the effect size with a confidence interval.	
Abstract	11 Provide an accurate summary of the research objectives, animal species, strain and sex, key methods, principal findings, and study conclusions.	11 We engrafted human immune cells in three different concentrations into immunodeficient female NOD/Shi-Prkdc^{scid}*Il2rg*^{em1}/Cyagen mice. Then, the initial points of development of a severe graft-versus-host reaction and the maximum possible time window for humane observation were determined. The study included regular complete blood count and the monitoring of the dynamics of the concentration of human cells in the blood of mice. In addition, the effect of grafts on the activation of the recipient's immune system was assessed. Finally, necropsy and histological and immunohistochemical examinations of the organs were performed to determine the localization of human cells. In this way, critical factors determining the success of human immune system reconstitution in mice were identified.<br>Myeloablation and the type and concentration of the graft are critical in the development of humanized immune system C-NKG mouse models. Proper irradiation dose and graft concentration result in prolonged circulation of HCs in mice. This is achieved by delaying the onset of GVHD, extending "the observation window". Overall, 2.5×10^6 hPBMCs and 2.5×10^6 hT-cells/hPBMCs mice showed engraftment rates of ~55 - 77% and ~66 - 78%, respectively, at 82 d.p.i. However, 82 days is not the limit of observation for these mice.<br>hCD4$^+$-cell infiltrates were detected in BM, perivascular interstitium of the liver and kidneys, splenic parenchyma, perivascular and peribronchial interstitium of the lungs, lamina propria of the small intestine, endometrium, brain, and mesenteric lymph nodes.<br>We found a high positive correlation between:<br>- the degree of chimerism and GVHD progression in 2.5×10^6 hPBMCs and 2.5×10^6 hT-cells/hPBMCs mice;<br>- hT-helper cell concentration and GVHD progression in mice 10×10^6 hPBMCs, 5×10^6 hT-cells/hPBMCs and 10×10^6 hT-cells/hPBMCs;<br>- GVHD progression and WBC including Lymph# in 10×10^6 hPBMCs mice;<br>- the degree of chimerism and WBC including Lymph# in 10×10^6 hPBMCs and 5×10^6 hT-cells/hPBMCs mice;<br>- the degree of chimerism and Mon# in hPBMCs and 5×10^6 hPBMCs mice;<br>- the degree of chimerism and Gran# in hPBMCs and 5×10^6 hT-cells/hPBMCs mice.

		High negative correlations were found between:<br>- GVHD progression and RBC in all humanized mice;<br>- GVHD progression and PLT in $10×10^6$ hPBMCs and $10×10^6$ hT-cells/hPBMCs mice;<br>- the degree of chimerism and RBC in $5×10^6$ hPBMCs and $10×10^6$ hT-cells/hPBMCs mice;<br>- the degree of chimerism and PLT in $10×10^6$ hPBMCs and $10×10^6$ hT-cells/hPBMCs mice.
Background	12 a. Include sufficient scientific background to understand the rationale and context for the study, and explain the experimental approach.<br>b. Explain how the animal species and model used address the scientific objectives and, where appropriate, the relevance to human biology.	12 a. The main challenge after engraftment of human tissues to mice is the development of graft-versus-host disease. It often occurs in an acute form, which reduces the time frame for observations. At the same time, different types of grafts can cause specific pathologies.<br>b. This is especially important to take into account when planning long-term studies of chronic diseases such as HIV infection. In addition, in mice, even with a similar genotype but different origin, the interaction between the graft and the recipient's organism can manifest itself differently.
Objectives	13 Clearly describe the research question, research objectives and, where appropriate, specific hypotheses being tested.	13 The aim of the study was to compare the effects of engraftment of hPBMCs and hT-cells mixed with autologous hPBMCs in NOD/Shi-*Prkdc*^{scid}*Il2rg*^{em1}/Cyagen mice over long time to determine the suitability of humanized mice for HIV infection.
Ethical statement	14 Provide the name of the ethical review committee or equivalent that has approved the use of animals in this study, and any relevant license or protocol numbers (if applicable). If ethical approval was not sought or granted, provide a justification.	14 The study was approved by the Central Research Institute of Epidemiology Commission for the Care and Scientific Purposes Use of Animals (CRIECCSPUA, Statement No. 2-HIS of 14.02.2024).
Housing and husbandry	15 Provide details of housing and	The animals were housed in the ISOcage P bioexclusion system (Techniplast, Italy) at a temperature of +21...+23

	husbandry conditions, including any environmental enrichment.	°C and an air humidity of 45 - 65%. Each cage was equipped with an igloo-type shelter. Animals were maintained under a 12-hour day/night cycle. Feeding was carried out with a standard maintaining diet for rodents (4RF21, Mucedola, Italy). The drinking was carried out with filtered tap water. Mice had unlimited access to water and feed. Fine-grained (3 mm fraction) birch chips (Russia) were used as bedding. Feed, drinking water and bedding were sterilized in an autoclave before being given to the animals. To enrich the environment, sterilized cardboard cups were used, which were replaced with new ones as they were chewed.
Animal care and monitoring	16 a. Describe any interventions or steps taken in the experimental protocols to reduce pain, suffering and distress.<br>b. Report any expected or unexpected adverse events.<br>c. Describe the humane endpoints established for the study, the signs that were monitored and the frequency of monitoring. If the study did not have humane endpoints, state this.	16 a. All mice were irradiated in a CIX3 X-ray irradiator cabinet (Xstrahl LTD, UK) one day prior to engraftment. Mice were previously anesthetized by inhalation of Isoflurane (Laboratorios Karizoo, Spain) using a Biosthesia 300 system (Vilber Lourmat, France). At the date of euthanasia or the humane endpoints were reached, the mice were anesthetized by intraperitoneal injection of Xylanite (90507, Nita-Pharm LLC, Russia) and Zoletil 100 (94123, Valdepharm, France) mixture. Euthanasia was performed by total exsanguination.<br>b. Not applicable.<br>c. Humane endpoints were defined:<br>- assigning a mouse 7 or more scores;<br>- body weight loss >25%.<br>The signs that were monitored twice-weekly:<br>- mouse activity;<br>- body weight;<br>- mouse posture;<br>- fur texture;<br>- skin condition.
Interpretation/ scientific implications	17 a. Interpret the results, taking into account the study objectives and hypotheses, current theory and other relevant studies in the literature.<br>b. Comment on the study limitations including potential sources of bias, limitations of the animal model, and imprecision associated with the results.	17 a. See the Table 12 of the manuscript.<br>b. We completed the study at 82 D.P.I. according to the study plan, however, we could continue observations for groups that received the minimum number of transplants. We expect that human cells can be infected with HIV by intravenous administration, but we do not know whether our models will be susceptible to vaginal and rectal infection.

Generalisability/ translation	18 Comment on whether, and how, the findings of this study are likely to generalize to other species or experimental conditions, including any relevance to human biology (where appropriate).	18 We believe that our results can be used in translational biomedical transfusion studies in the assessment of GVHD in humans. In addition, our data can be useful in evaluation double-humanized mice.
Protocol registration	19 Provide a statement indicating whether a protocol (including the research question, key design features, and analysis plan) was prepared before the study, and if and where this protocol was registered.	19 The protocol was prepared prior to the study, registered by the Institute's Quality Assurance and Archiving Group, and approved by the Central Research Institute of Epidemiology Commission for the Care and Scientific Uses of Animals (CRIECCSPUA, Statement No. 2-HIS of 02/14/2024).
Data access	20 Provide a statement describing if and where study data are available.	20 The study data are available in the text of the manuscript and supplementary materials.
Declaration of interests	21 a. Declare any potential conflicts of interest, including financial and non-financial. If none exist, this should be stated.<br>b. List all funding sources (including grant identifier) and the role of the funder(s) in the design, analysis and reporting of the study.	21 a. The authors declare no conflicts of interest.<br>b. This research was funded by the Ministry of Science and Higher Education of the Russian Federation within the framework of a grant in the form of a subsidy for the creation and development of the «World-class Genomic Research Center for Ensuring Biological Safety and Technological Independence under the Federal Scientific and Technical Program for the Development of Genetic Technologies», agreement № 075-15-2025-517. The funders had and will not have a role in study design, data collection and analysis, decision to publish, or preparation of the manuscript.
